# Solution‐Based 3D Printing of Thermoelectrics: Advances and Opportunities

**DOI:** 10.1002/advs.202514259

**Published:** 2025-11-03

**Authors:** Minzhi Du, Xiao‐Lei Shi, Yong Du, Ting Zhang, Zhi‐Gang Chen

**Affiliations:** ^1^ Faculty of Materials Technology Shanghai Institute of Technology 100 Haiquan Road Shanghai 201418 P. R. China; ^2^ School of Chemistry and Physics ARC Research Hub in Zero‐emission Power Generation for Carbon Neutrality and Centre for Materials Science Queensland University of Technology Brisbane QLD 4000 Australia; ^3^ Nanjing Institute of Future Energy System Nanjing 211135 P. R. China; ^4^ Institute of Engineering Thermophysics Chinese Academy of Sciences Beijing 100190 P. R. China; ^5^ University of Chinese Academy of Sciences Beijing 100049 P. R. China; ^6^ University of Chinese Academy of Sciences Nanjing 211135 P. R. China

**Keywords:** additive manufacturing, solution 3D printing, thermoelectrics

## Abstract

Conformal and flexible thermoelectric devices, capable of adapting to complex heat sources, are essential for advancing practical thermoelectric applications in energy harvesting and thermal management. Solution‐based 3D printing has emerged as a promising additive manufacturing technique for thermoelectrics, offering advantages such as ambient‐temperature processing, geometric precision, material versatility, and scalability for mass customization. This review highlights recent progress in conformal and flexible thermoelectric materials and devices fabricated via solution 3D printing, with a systematic focus on ink formulations, material and device architectures, performance metrics, and real‐world applications. It also discusses current challenges, future directions, and emerging opportunities. This work aims to inform the design and scalable fabrication of high‐performance, flexible, and adaptable thermoelectric systems.

## Graphical abstract

Solution 3D printing presents a versatile platform for fabricating conformal and flexible thermoelectric devices, enabling efficient thermal energy harvesting. This review summarizes recent advancements in thermoelectric inks, materials, device architectures, performance metrics, and practical applications, while also addressing current challenges and future opportunities toward the development of high‐performance, adaptable thermoelectric technologies.

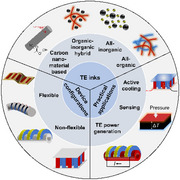

## 1 Introduction

Thermoelectrics serve dual roles as thermoelectric generators (TEGs), which convert waste heat into electricity, and thermoelectric coolers (TECs), which provide cooling through direct current input.^[^ ^1^, ^2^, ^3^, ^4^, ^5^ ^]^ With the rising demand for Internet‐of‐Things (IoT) devices and wearable technologies,^[^ ^6^, ^7^, ^8^, ^9^ ^]^ thermoelectrics have gained attention for sustainable applications such as self‐powered electronics,^[^ ^10^, ^11^ ^]^ battery‐less sensors,^[^ ^12^, ^13^ ^]^ on‐chip cooling,^[^ ^14^, ^15^ ^]^ and personal thermoregulation.^[^ ^16^, ^17^ ^]^ At the material level, thermoelectric performance is defined by the dimensionless figure of merit, *ZT* = *S* ^2^ *σT*/*κ*, where *S* is the Seebeck coefficient, *σ* is electrical conductivity, *T* is temperature, and *κ* is total thermal conductivity. At the device level, the maximum power conversion efficiency (*η*_max_) of TEGs depends on the temperature gradient and the average *ZT* of the constituent materials,^[^ ^18^, ^19^ ^]^ while TEC efficiency is governed by the coefficient of performance (*COP*).^[^ ^20^, ^21^ ^]^ Thus, achieving high *ZT* values in conjunction with optimized device architectures is crucial for maximizing thermoelectric energy conversion.

Conformal and flexible thermoelectric devices (TEDs) hold great promise for efficient thermal energy harvesting and heat transfer due to their adaptability to complex heat sources and reduced thermal losses.^[^ ^5^ ^]^ Conventional thermoelectric materials, typically bismuth‐, antimony‐, and lead‐based tellurides, are fabricated using techniques such as ball milling, hot pressing, spark plasma sintering, and zone melting.^[^ ^3^, ^22^, ^23^ ^]^ However, these methods involve rigid structures, high energy consumption, and prolonged processing at elevated temperatures, posing major barriers to the development of flexible and conformal TEDs.^[^ ^24^ ^]^ To overcome these challenges, advanced manufacturing strategies are urgently required to improve scalability, enhance system‐level performance, and lower production costs, thereby accelerating the commercial deployment of thermoelectric technologies.^[^ ^25^ ^]^

Additive manufacturing (AM), commonly known as 3D printing, enables the fabrication of diverse, customized products from computer‐generated models and has emerged as a transformative force in modern manufacturing.^[^ ^24^ ^]^ Among various AM techniques such as stereolithography (SLA), fused deposition modeling (FDM), selective laser melting/sintering (SLM/SLS), and solution 3D printing (SP),^[^ ^26^, ^27^, ^28^ ^]^ solution 3D printing stands out for its exceptional versatility. It supports a wide range of solution‐based inks, operates at ambient temperature, consumes less energy, and offers high flexibility.^[^ ^29^ ^]^ These advantages make solution 3D printing one of the most practical AM methods, particularly well‐suited for fabricating conformal and flexible thermoelectric materials and devices.

**Figure 1:**
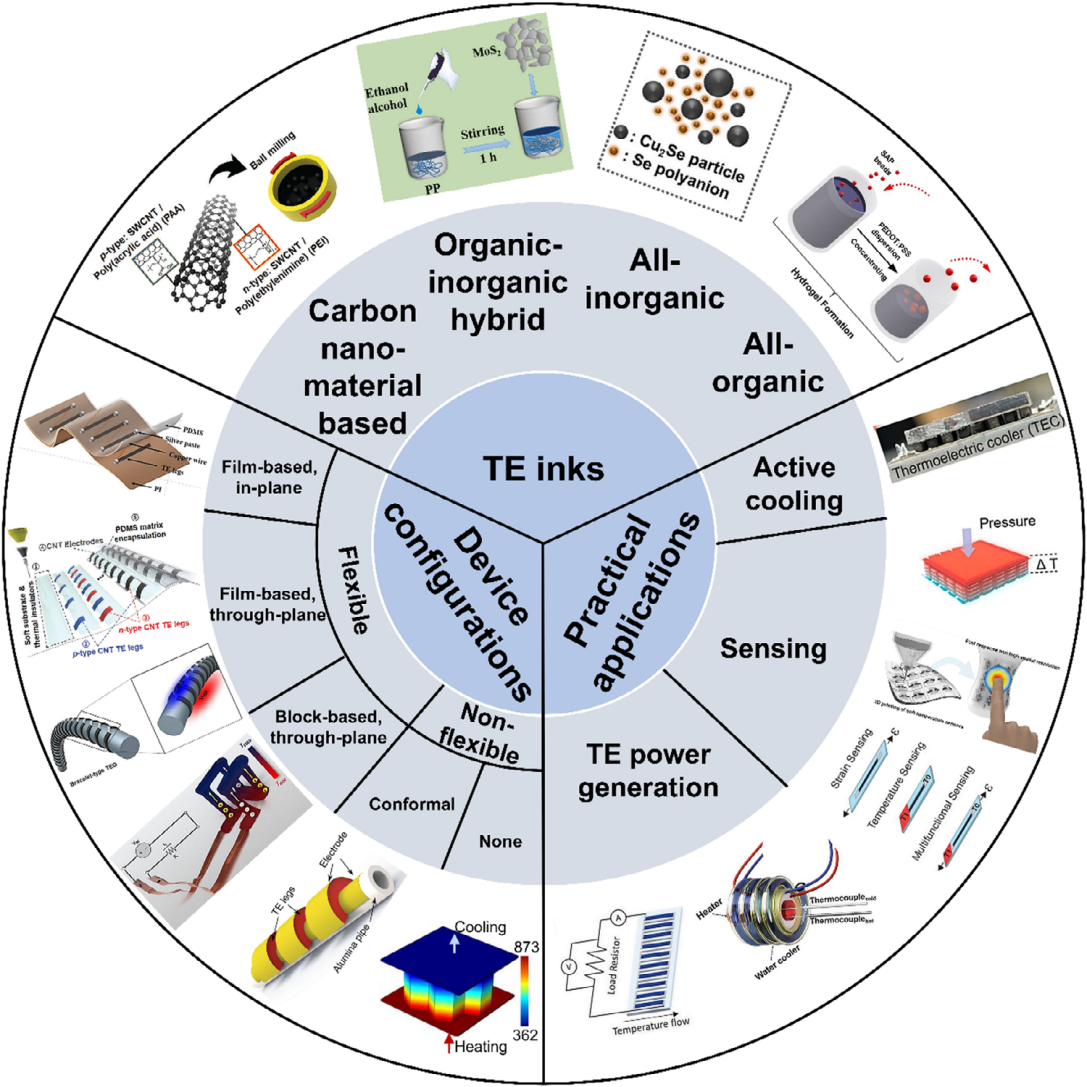
Representative examples of thermoelectric slurries, device configurations fabricated via solution‐based 3D printing, and their corresponding applications.^[^ ^30^, ^31^, ^32^, ^33^, ^34^, ^35^, ^36^, ^37^, ^38^, ^39^, ^40^, ^41^, ^42^, ^43^ ^]^ Counterclockwise from All‐organic ink: Reproduced with permission.^[^ ^30^ ^]^ Copyright 2023, Elsevier. Reproduced with permission.^[^ ^31^ ^]^ Copyright 2021, Springer Nature. Reproduced with permission.^[^ ^32^ ^]^ Copyright 2021, Elsevier. Reproduced with permission.^[^ ^33^ ^]^ Copyright 2023, Wiley‐VCH. Reproduced with permission.^[^ ^34^ ^]^ Copyright 2024, American Chemical Society. Reproduced with permission.^[^ ^35^ ^]^ Copyright 2018, Royal Society of Chemistry. Reproduced with permission.^[^ ^36^ ^]^ Copyright 2024, Royal Society of Chemistry. Reproduced with permission.^[^ ^37^ ^]^ Copyright 2022, Royal Society of Chemistry. Reproduced with permission.^[^ ^38^ ^]^ Copyright 2019, Wiley‐VCH. Reproduced with permission.^[^ ^39^ ^]^ Copyright 2021, Wiley‐VCH. Reproduced with permission.^[^ ^40^ ^]^ Copyright 2024, IEEE. Reproduced with permission.^[^ ^41^ ^]^ Copyright 2024, American Chemical Society. Reproduced with permission.^[^ ^43^ ^]^ Copyright 2021, Elsevier. Reproduced with permission.^[^ ^42^ ^]^ Copyright 2025, American Association for the Advancement of Science.

This review highlights recent advancements in solution‐based 3D printing for thermoelectric materials and devices. It begins with a brief introduction to solution 3D printing technology, followed by a detailed examination of thermoelectric ink formulations, flexible material and device configurations, performance metrics, and practical applications (**Figure** **1**). The review also addresses current challenges, prospects, and emerging directions in the field, aiming to provide valuable guidance for the design and scalable fabrication of high‐performance, flexible, and conformal TEDs.

## 2 Fundamentals of Thermoelectrics

### 2.1 Thermoelectric Figure of Merit

TEDs typically consist of multiple thermocouples, each formed by *p*‐type and *n*‐type materials that are electrically connected in series and thermally in parallel. Consequently, the overall conversion efficiency (*η*) of TEDs largely depends on the thermoelectric properties of the constituent materials, commonly evaluated by the *ZT* defined in Equation (1).^[^ ^22^ ^]^ The *S* ^2^ *σ* refers to the power factor (*PF*), which is a key parameter for evaluating thermoelectric performance.

(1) $$ZT=\frac{S^{2}\sigma T}{\kappa }$$

For inorganic thermoelectric materials, *S*, 𝜎, and *κ* can be expressed by Equations (2), (3), and (4), wherein *k*_B_, *e*, *h*, *m* ^*^, *n*, *μ*, *κ*_e_, and *κ*_l_ are the Boltzmann constant, elementary charge, Planck constant, carrier effective mass, carrier concentration, carrier mobility, electrical and lattice thermal conductivity, respectively.^[^ ^44^ ^]^

(2) $$S=\frac{8\pi^{2}k_{B}^{2}}{3eh^{2}}m^{*}T{\left(\frac{\pi }{3n}\right)}^{2/3}$$

(3) σ=neμ

(4) $$\kappa =\kappa_{e}+\kappa_{l}$$

Achieving high thermoelectric performance requires materials that simultaneously exhibit a high *S* and *σ*, along with low *κ*, to maximize the *ZT*. However, this is inherently challenging due to the intrinsic trade‐offs among these parameters: *σ* and *κ* are typically positively correlated, while *S* and *σ* generally show an inverse relationship in most thermoelectric materials.^[^ ^45^, ^46^ ^]^

### 2.2 Output Performance of TEDs

In the power generation mode of TEGs, the open‐circuit voltage (*V*_oc_) under a given temperature difference (Δ*T*) is calculated using Equation (5), where *S*_p_ and *S*_n_ are the absolute Seebeck coefficients of the *p*‐type and *n*‐type legs, respectively, and *N* is the number of thermocouples.^[^ ^13^ ^]^

(5) $$V_{\mathrm{oc}}=\left(S_{p}+S_{n}\right)\Delta T\times N$$

When a TEG with a certain internal resistance (*r*) is connected with an external load resistance (*R*), the circuit current (*I*), output voltage (*V*_output_), and output power (*P*_output_) can be expressed as Equations (6), (7), and (8), respectively.^[^ ^13^ ^]^

(6) $$I=\frac{V_{\mathrm{oc}}}{r+R}=\frac{\left(S_{p}+S_{n}\right)\Delta T\times N}{r+R}$$

(7) $$V_{\mathrm{output}}=IR$$

(8) $$P_{\mathrm{output}}=I^{2}R={\left[\frac{\left(S_{p}+S_{n}\right)\Delta T\times N}{r+R}\right]}^{2}R$$

At a given Δ*T*, the maximum output power (*P*_max_) is achieved when electrical matching is satisfied (*R* = *r*). This condition is expressed by Equation (9).

(9) $$P_{\max}=\frac{{V_{\mathrm{oc}}}^{2}}{4R}=\frac{{\left[\left(S_{p}+S_{n}\right)\Delta T\times N\right]}^{2}}{4R}$$

Additionally, the output power density (*P*_d_) is used to compare the normalized output performance. It is calculated as the output power divided by the total surface area (*A*) of the TEG, as shown in Equation (10).^[^ ^47^ ^]^

(10) $$P_{d}=\frac{P}{A}$$

In the cooling mode of TECs, the cooling capacity (*Q_c_*) and the attainable Δ*T* are critical parameters for assessing the cooling effect. These parameters are defined by following Equations (11) and (12), respectively.^[^ ^16^, ^17^, ^48^ ^]^

(11) $$Q_{C}=S_{\mathrm{TEC}}\left(T_{h}-\Delta T\right)I-\frac{1}{2}I^{2}R-K\cdot \Delta T$$

(12) $$\Delta T=\frac{S_{\mathrm{TEC}}T_{c}I-\frac{1}{2}I^{2}R-Q_{c}}{K}$$

Herein *S*_TEC_ is the Seebeck coefficient of the whole device, *T*_h_ and *T*_c_ are the temperatures of hot and cold sides, and *K* is the thermal conductance of TEC.

### 2.3 Maximum Energy Conversion Efficiency

For TEGs, the *η*_max_ is expressed by Equation (13),^[^ ^49^ ^]^ where *ZT*_avg_ is the materials’ average figure of merit. Heat loss in Joule heating, heat evaporation, and other irreversible processes occur due to the parasitic effect, the conversion efficiency of TEGs is far below Carnot efficiency ($\eta_{c}=\frac{T_{h}-\;T_{c}}{T_{h}}$).

(13) $$\eta_{\max}=\frac{T_{h}-\;T_{c}}{T_{h}}\frac{\sqrt{1+ZT_{\mathrm{avg}}}\;-\;1}{\sqrt{1+ZT_{\mathrm{avg}}}+\frac{T_{c}}{T_{h}}}$$

For TECs, the energy conversion efficiency is estimated by the *COP*. The maximum *COP* is defined as Equation (14).^[^ ^15^ ^]^

(14) $$COP_{\max}=\frac{T_{c}}{T_{h}-\;T_{c}}\frac{\sqrt{1+ZT_{\mathrm{avg}}}\;-\;\frac{T_{h}}{T_{c}}}{\sqrt{1+ZT_{\mathrm{avg}}}+1}$$

In abovementioned *η*_max_ and *COP*_max_, *ZT*_avg_ can be described as Equation (15).^[^ ^50^, ^51^ ^]^

(15) $$ZT_{\mathrm{avg}}=\frac{\int_{T_{c}}^{T_{h}}ZTdT}{T_{h}-\;T_{c}}$$

## 3 Solution 3D Printing

### 3.1 Technological Process

**Figure 2:**
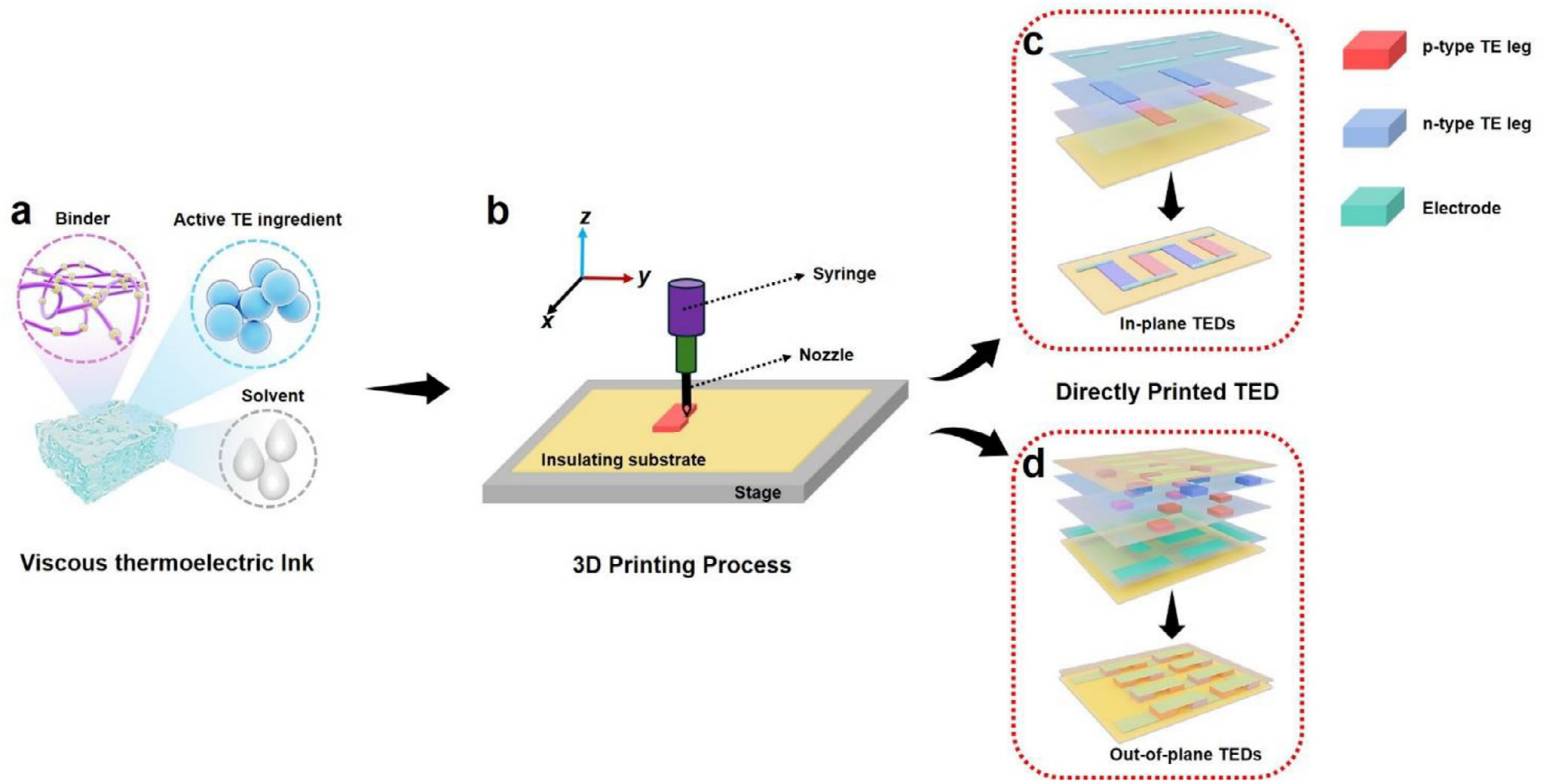
Technological process diagram of solution 3D printing. Illustration of a) viscous thermoelectric inks, b) 3D printing process, directly printed thermoelectric devices (TEDs) including c) in‐plane and d) out‐of‐plane TEDs.

Solution 3D printing, also referred to as material deposition, extrusion‐based 3D printing, or direct ink writing (DIW), is a widely adopted bottom‐up additive manufacturing technique known for its cost‐effectiveness, versatility, and broad material compatibility.^[^ ^52^ ^]^ As illustrated in **Figure** **2**, the process comprises several key steps: 1) Thermoelectric Ink Preparation (Figure 2a): Selection of thermoelectric active materials and formulation of homogeneous, rheological inks using appropriate binders and dispersion solvents; 2) Ink Deposition (Figure 2b): Controlled extrusion of thermoelectric inks through a nozzle or dispenser under defined pressure and temperature, typically onto insulating substrates such as nylon membranes or polyimide films; 3) Post‐Treatment: Processing steps including pre‐curing, cold/hot pressing, and annealing to form planar thermoelectric films or 3D thermoelectric blocks; 4) TED Fabrication (Figure 2c,d): TEDs can be assembled from prefabricated thermoelectric strips or blocks with internal electrode connections, or printed directly with integrated electrode arrays and alternating p‐/n‐type thermoelectric segments, followed by post‐processing to enhance performance; 5) Encapsulation and Application: Final encapsulation prepares the device for practical heat‐to‐electricity conversion. This streamlined process offers a scalable and efficient route for fabricating high‐performance, flexible TEDs for a wide range of applications.

### 3.2 Comparison for Solution 3D Printing With Other AM Processes

When comparing AM processes for thermoelectrics, each technique exhibits distinct trade‐offs. Stereolithography (SLA)^[^ ^50^, ^53^, ^54^, ^55^ ^]^ employs ultraviolet lasers to cure photosensitive resins containing thermoelectric powders layer by layer. This method offers excellent spatial resolution (<100 µm) for fabricating intricate geometries, but its limited thermoelectric filler loading and curing‐related defects (e.g., interlayer delamination, pores, and cracks from incomplete or over‐curing of insulating resins) lead to high electrical resistivity and poor device performance. SLA is particularly suitable for rapid prototyping of complex structures; however, it is time‐consuming, costly, and requires additional washing and post‐curing steps, restricting its scalability for large‐area TED fabrication. For example, He et al. ^[^ ^53^ ^]^ prepared a p‐type thermoelectric bulk using SLA with a slurry of 60 wt% Bi_0.5_Sb_1.5_Te_3_ particles in photoresins, followed by annealing at 350 °C for 6 h to decompose the resin. The decomposition generated amorphous carbon, which increased porosity and resulted in an inferior *ZT* of 0.12 at 43 °C.

Fused deposition modeling (FDM)^[^ ^26^, ^50^, ^56^ ^]^ or thermoelectrics involves heating and extruding composite filaments of thermoelectric fillers blended with thermoplastic polymers (e.g., ABS, PLA), which are then deposited layer by layer to form the designed structures. This technique offers a simple and low‐cost route for fabricating flexible or geometrically complex prototypes. However, the process inherently produces high porosity and anisotropic structures due to its layer‐by‐layer extrusion, while weak interlayer bonding and warping arise from uncontrolled cooling and shrinkage. As a result, FDM‐printed TEDs generally exhibit poor mechanical integrity and extremely low conversion efficiency. For instance, AW et al. ^[^ ^57^ ^]^ fabricated acrylonitrile–butadiene–styrene/zinc oxide (ABS/ZnO) composites by FDM, where the as‐printed sample with 14 wt% ZnO achieved a *ZT* of only ≈5.7 × 10^−5^. Moreover, FDM typically offers low spatial resolution (>200 µm), which restricts its utility in high‐performance device fabrication, although it provides good efficiency for prototyping and the potential for large‐scale production.

Selective laser melting/sintering (SLM/SLS)^[^ ^50^, ^55^, ^58^ ^]^ employs high‐energy lasers to selectively melt or sinter thin layers of thermoelectric powders (e.g., polymers or inorganic compounds), thereby forming 3D solids without the need for substrates. This technique enables the direct fabrication of dense, high‐performance thermoelectric alloy components with high resolution (20–100 µm) and fast printing speeds (50–500 mm s^−1^). For instance, Qiu et al. ^[^ ^59^ ^]^ reported a Bi_0.4_Sb_1.6_Te_3_ bulk fabricated by SLM with a *ZT* of 1.1 at 316 K, while Shi et al. ^[^ ^58^ ^]^ prepared a porous Bi_0.5_Sb_1.5_Te_3_ bulk using SLS, achieving a *ZT* of ≈1.29 at 54 °C. Nevertheless, for brittle thermoelectric semiconductors, issues such as cracking, warping, and void formation commonly arise from high thermal stresses, while elemental segregation induced by high‐energy laser irradiation further degrades thermoelectric performance. In addition, the high cost of equipment and raw materials, the need for inert atmospheres, and time‐intensive pre‐ and post‐processing steps significantly limit the scalability of SLM/SLS for thermoelectric material and device manufacturing.

Solution 3D printing (SP)^[^ ^19^, ^54^, ^60^, ^61^ ^]^ offers remarkable versatility in material accessibility, enabling the fabrication of high‐solid‐loading, polymer‐free thermoelectric inks for high‐performance devices. However, defects such as cracking, porosity, and weak interlayer interfaces often arise during drying and sintering, primarily due to ink bubbles, particle agglomeration, solvent evaporation, and uneven shrinkage. The spatial resolution of SP is moderate (>100 µm), constrained by nozzle diameter. Benefiting from its continuous deposition process, SP provides relatively high scalability and manufacturing efficiency. Importantly, the thermoelectric performance of SP‐fabricated devices can rival that of bulk materials, but it strongly depends on precise ink formulation and carefully optimized post‐treatments to minimize defects and ensure full densification.

To address the challenges in solution 3D printing, multi‐faceted strategies are required. The use of dispersants, thixotropic agents, adhesives, and plasticizers can effectively tune ink rheology, ensuring uniform flow and preventing filler sedimentation. Controlled drying (either under relatively humid conditions or through a staged approach with gradually increasing temperature) reduces solvent evaporation rates, lowers capillary forces, and suppresses cracking. Isostatic cold pressing prior to annealing or sintering can further mitigate porosity and cracking in printed structures. Additionally, interface engineering strategies, such as interlayer redissolution and co‐sintering, can strengthen interlayer adhesion and prevent delamination in multi‐material systems.

**Table** **1** summarizes the key distinctions among various AM techniques used in thermoelectric fabrication, covering their working principles, compatible material systems, resolution and printing speed, typical defect types and formation mechanisms, as well as their respective advantages and limitations in producing thermoelectric materials and device prototypes.

**Table 1:** Comparison of typical AM techniques applied to the fabrication of thermoelectric materials and device prototypes.

AM technique	Operational principle	Applicable material	Resolution and speed	Defect type	Defect formation	Superiorities	Limitations	Manufacturing efficiency	Reference.
SLA	Photocurable polymer resin solidified layer‐by‐layer when exposed to the ultraviolet laser according to the computer‐controlled instructions	Hybrid of photocurable resins and thermoelectric powders	Resolution <100 µm, speed <20 mm s^−1^	Delamination, pores & cracks	Improper control of UV penetration depth, agglomerated fillers or air bubbles in slurries	Achieving any self‐supporting geometry with high‐resolution	Limited photo‐resin, porous internal structure and ribbed surface of printed materials, etc.	Low‐Medium	[50, 53, 54, 55]
FDM	Computer‐designed structures obtained from thermoplastic composite filaments’ melting and depositing layer by layer	Composite filaments of thermoelectric fillers/thermoplastic polymers (ABS, PLA, etc.)	Resolution >100 µm, speed <20 mm s^−1^	High porosity, anisotropic structure, layer lines, etc.	Inherence of the layer‐by‐layer extrusion process and uncontrolled cooling and shrinkage	Substrate‐free	Complex coordination process, high‐temperature process, etc.	Medium	[26, 50, 56]
SLM/SLS	Computer‐designed structures obtained from powders sintering with selective high‐energy lasers	Polymer powders, metal powders, inorganic thermoelectric powders, etc.	Resolution >30 µm, speed 50–500 mm s^−1^	Cracking, warping, spiracles, element segregation, etc.	High thermal stress and improper laser power/scanning strategy	Substrate‐free, high resolution, etc.	High cost, time‐consuming, etc.	Low‐Medium	[50, 55, 58]
SP	Solution‐based deposition process according to the computer‐controlled instructions	Rheological solutions containing thermoelectric materials	Resolution >100 µm, speed <20 mm s^−1^	Cracking, pores, interlayer interfaces, etc.	Rheological instability, solvent evaporation and uneven shrinkage	Applicability for any rheological solution/paste	Shrinkage	Medium	[19, 54, 60, 61]

: ABS = acrylonitrile butadiene styrene and PLA = polylactic acid.

### 3.3 Type of Thermoelectric Inks

Thermoelectric inks or slurries are a critical component of solution 3D printing, and their rheological properties must be carefully tailored to ensure printing success. In this process, inks are extruded through a nozzle mounted on an *x*–*y*–*z* motion stage to construct multidimensional structures. Key requirements for thermoelectric inks include appropriate viscosity, good fluidity, and excellent component stability:

1. **Rheology**: Inks with optimized rheological properties ensure consistent line definition during nozzle movement, minimizing spreading on the substrate and preserving structural integrity throughout the printing, curing, and sintering processes.^[^ ^61^ ^]^
2. **Fluidity**: Shear‐thinning behavior is essential for smooth extrusion under pressure, allowing the ink to flow easily through the nozzle while ensuring continuous and uniform deposition.^[^ ^62^ ^]^
3. **Component Stability**: Long‐term stability ensures uniform dispersion of all components, enabling consistent ink performance during both storage and printing.

To satisfy these criteria, a variety of material systems and ink formulation strategies have been developed and optimized for thermoelectric applications.

#### 3.3.1 All‐Organic Thermoelectric Inks

In formulating thermoelectric inks, conductive polymers and their derivatives, particularly poly(3,4‐ethylenedioxythiophene):poly(styrenesulfonate) (PEDOT:PSS), are commonly used as primary thermoelectric active components.^[^ ^63^ ^]^ For example, Shakeel et al.^[^ ^64^ ^]^ employed pristine PEDOT:PSS directly as the printing ink. To increase ink viscosity and enhance the mechanical robustness of printed structures, macromolecular polymers dissolved in suitable solvents are often introduced as binders. Li et al.^[^ ^30^ ^]^ incorporated superabsorbent polymer (SAP) beads into PEDOT:PSS dispersions to formulate printable inks. Since high electrical conductivity is essential for thermoelectric performance, secondary doping agents are frequently added.^[^ ^38^, ^40^, ^65^ ^]^ For instance, Kee et al.^[^ ^38^ ^]^ developed a ternary composite by blending PEDOT:PSS with the polymeric surfactant Triton X‐100 (as a healing agent) and dimethyl sulfoxide (DMSO) to enhance thermoelectric properties. These examples illustrate the diverse strategies used to optimize TE ink compositions for solution 3D printing.

#### 3.3.2 All‐Inorganic Thermoelectric Inks

Inorganic thermoelectric materials, especially Bi_2_Te_3_‐, Ag_2_Se‐, and Mg_3_Bi_2_‐based near‐room‐temperature thermoelectric materials, generally exhibit superior performance compared to their organic counterparts.^[^ ^66^, ^67^, ^68^, ^69^, ^70^, ^71^, ^72^ ^]^ All‐inorganic thermoelectric inks, compatible with solution 3D printing, enable the fabrication of conformal TEDs for efficient and adaptable waste heat harvesting. The preparation process typically begins with high‐energy ball milling to reduce thermoelectric granules or powders to fine particles, followed by sieving to remove agglomerates. The refined particles are then dispersed in organic solvents, such as ethylene glycol (EG), glycerol, or ethanol, and homogenized via centrifugal mixing to form printable inks.^[^ ^31^, ^34^, ^39^, ^73^, ^74^, ^75^, ^76^ ^]^ To prevent oxidation of the inorganic components, these steps are often carried out under an inert atmosphere. For instance, Kim et al.^[^ ^73^ ^]^ prepared Bi_2_Te_3_‑based all‐inorganic inks by ball‐milling Bi, Sb, Te, and Se powders under a nitrogen atmosphere, followed by homogenizing the mixture with Sb_2_Te_4_ chalcogenidometallate (ChaM) in glycerol. The performance of all‐inorganic thermoelectric inks can be further enhanced by tailoring the chemical composition and structural features of the thermoelectric components.^[^ ^31^, ^39^, ^73^, ^74^, ^75^, ^76^ ^]^ Kim *et al.*,^[^ ^75^, ^76^ ^]^ for example, developed a high‐performance (Bi, Sb)_2_(Te, Se)_3_‐based ink by combining ball‐milled thermoelectric particles with Sb_2_Te_4_ ChaM, which also functioned as a binder. These developments underscore the strong potential of all‐inorganic thermoelectric inks for fabricating high‐efficiency TEDs via solution 3D printing.

#### 3.3.3 Organic/Inorganic Hybrid Thermoelectric Inks

Organic/inorganic hybrid thermoelectric inks combine the favorable viscosity and rheological properties of polymer‐based solutions with the functional advantages of inorganic fillers, making them highly suitable for fabricating high‐performance flexible or conformal TEDs. PEDOT:PSS is frequently employed as both a conductive matrix and an active thermoelectric component.^[^ ^77^ ^]^ For example, Xu et al.^[^ ^32^ ^]^ prepared MoS_2_/PEDOT:PSS composite slurries using MoS_2_ as the filler and PEDOT:PSS as the matrix, and similarly formulated black phosphorus/PEDOT:PSS inks with 5 vol% DMSO to enhance performance.^[^ ^78^ ^]^ Insulating polymers such as polylactide‐co‐glycolide (PLG), polylactic acid (PLA), polyvinylpyrrolidone (PVP), polystyrene (PS), and methylcellulose (MC) are commonly used as binders in hybrid thermoelectric inks. Du et al.,^[^ ^79^ ^]^ for instance, developed a ternary thermoelectric ink by dissolving Bi_0.4_Sb_1.6_Te_3_ and carbon black into a PLA/chloroform (CLM) solution, a strategy similarly employed across various material systems.^[^ ^80^, ^81^, ^82^, ^83^ ^]^ To further tune ink properties, plasticizers and surfactants are often incorporated.^[^ ^84^, ^85^, ^86^ ^]^ Peng et al.^[^ ^84^ ^]^ refined a Bi_2_Te_3_/PLG slurry via ball milling, adding 2‐butoxyethanol (BCS) as a surfactant and dibutyl phthalate (DBP) as a plasticizer. In addition, polyelectrolytes such as poly(acrylic acid) (PAA) and poly(ethylenimine) (PEI) have been used to regulate ink rheology and improve stability by modifying particle surface charge, as demonstrated in Bi_0.5_Sb_1.5_Te_3_/MC hybrid inks.^[^ ^37^, ^87^ ^]^ These examples underscore the versatility of organic/inorganic hybrid thermoelectric inks in tuning material properties and optimizing printability, enabling the scalable fabrication of advanced TEDs.

#### 3.3.4 Carbon Nano‐Material Based Thermoelectric Inks

Carbon nanomaterials, such as 1D carbon nanotubes (CNTs) and carbon nanofibers with high aspect ratios, have been extensively investigated for thermoelectric applications due to their excellent thermoelectric properties and mechanical durability.^[^ ^63^, ^68^, ^88^, ^89^, ^90^ ^]^ Importantly, CNTs can be readily tuned into *p*‐type or *n*‐type thermoelectric materials through the use of organic dopants.^[^ ^33^, ^91^, ^92^ ^]^ In the context of solution 3D printing, carbon nanomaterials and their composites offer significant advantages. They facilitate the formulation of highly concentrated inks with tunable viscosities and superior printability, making them ideal for fabricating advanced TEDs.

Park et al.^[^ ^35^ ^]^ directly formulated printable thermoelectric inks by dispersing single‐walled CNTs (SWCNTs) in diethylene glycol (DEG) via planetary ball milling. Polyelectrolytes, PAA and PEI were used as *p*‐ and *n*‐type dopants, respectively, and served as binders. Hwang et al.,^[^ ^33^, ^41^ ^]^ employed a similar approach to prepare PAA‐ and PEI‐doped SWCNT‐based thermoelectric inks. In addition to direct dispersion, carbon nanotubes can be combined with conducting polymers to enhance ink properties. For instance, Li et al.^[^ ^43^ ^]^ synthesized composite hydrogels of PEDOT:PSS and multi‐walled CNTs (MWCNTs) via a self‐assembled gelation process without surfactants. After DMSO immersion and freeze‐drying, the resulting hydrogel was redispersible in water and directly usable as a 3D printing ink. Insulating polymers have also been incorporated to improve print resolution and continuity. Yin et al.^[^ ^43^ ^]^ developed a printable thermoelectric ink by dispersing carbon nanofibers in a diluted thermoplastic polyurethane (TPU) solution through thorough mixing and defoaming. Similarly, Mytafides et al.^[^ ^36^ ^]^ prepared homogeneous *p*‐ and *n*‐type thermoelectric inks by dispersing PEDOT:PSS/SWCNTs and PEI/SWCNTs, respectively, into Superflex, a flexible epoxy resin. These strategies highlight the versatility of carbon nanomaterials and their composites in the development of high‐performance, printable thermoelectric inks for solution 3D printing.

### 3.4 Critical Challenges for Solution 3D Printing Process

Solution‐based 3D printing of thermoelectric materials faces significant challenges in both material formulation and process optimization, which directly influence printability and final device performance. From the perspective of ink formulation, particle characteristics such as size distribution and concentration, must strike a balance between flowability and functional performance. Large particles (>5 µm) increase the risk of nozzle clogging, while nanoparticles (<100 nm) are prone to agglomeration, leading to either clogging or non‐uniform printed structures. High solid loading is generally beneficial for enhancing thermoelectric performance but negatively impacts rheological behavior, whereas low particle concentrations result in excessive porosity after post‐treatment. To ensure printability, inks must exhibit suitable rheological properties, particularly shear‐thinning behavior with viscosities in the range of 10–100 Pa·s at 100 s^−1^, to enable smooth extrusion while rapidly recovering elastic modulus to maintain shape fidelity. Solvent selection further complicates formulation: high‐boiling‐point solvents (e.g., DMSO) slow drying and promote sedimentation, while low‐boiling‐point solvents (e.g., ethanol) may evaporate prematurely, causing nozzle clogging. Process parameters also require careful tuning. The nozzle diameter and printing speed must be optimized to balance resolution and reliability. Finer nozzles (<100 µm) improve feature resolution but are more susceptible to clogging. Conversely, printing speeds exceeding 20 mm s^−1^ may induce structural fractures, while speeds below 5 mm/s can lead to material over‐spreading. Post‐processing introduces additional complications. Organic components must be thermally decomposed without compromising structural integrity, while inorganic components are vulnerable to volatilization during high‐temperature sintering, for instance, Se or Te loss at temperatures above 400 °C. Moreover, drying shrinkage may result in crack formation, further affecting mechanical and functional performance. To address the challenges, several strategies can be employed, including the design of core–shell microstructures (e.g., PEDOT:PSS‐coated Cu_2_Se nanowires), the use of rheological modifiers (e.g., nanocellulose), and co‐solvent engineering (e.g., ethanol–glycerol blends). While these approaches involve inherent trade‐offs such as sacrificing thermoelectric performance (*ZT*) for improved printability, they enable the realization of key advantages unique to solution‐based 3D printing. These include the fabrication of conformal designs with reduced thermal contact resistance and flexible devices capable of adapting to non‐planar surfaces, thereby expanding the practical application potential of thermoelectric devices in complex geometries and wearable energy harvesting systems.

**Table** **2** presents a comprehensive summary of recently developed thermoelectric inks, outlining their precursors, functional components, binders, dispersion solvents, preparation methods, and processing conditions.

**Table 2:** Summary of the types, precursor, ingredients, binder, dispersion solvent, preparation process and conditions for thermoelectric inks.

Ink type	Precursor	Active ingredients	Other ingredients	Binder	Dispersion solvent	Preparation process and conditions	Refs.
	/	PEDOT:PSS	/	/	/	/	[64]
	/	DMSO‐added PEDOT:PSS	Triton^TM^ X‐100	/	/	Mixing and stirring at RT	[38]
All‐organic inks	/	DMSO‐added PEDOT:PSS	/	PLA	/	Mixing, heating at 95 °C, and stirring at RT	[65]
	/	PEDOT:PSS	SAP	/	/	Mixing and stirring at RT	[30]
	/	DMSO‐added PEDOT:PSS	Li salt and GOPS	/	Deionized water	Stirring at 60 °C	[93]
	/	DMSO‐added PEDOT:PSS	/	PEO	/	Stirring at 43 °C	[40]
	Granules of Bi, Sb, Te, and Se	Bi_0.4_Sb_1.6_Te_3_ and Bi_2_Sb_2.7_Se0._3_	/	Sb_2_Te_4_ ChaM	Glycerol	Ball milling and centrifugally mixing under a N_2_ atmosphere	[73]
	SbCl_3_ and TeO_2_	Sb_2_Te_3_	Te	/	Mixture of EG, glycerol, and ethanol	Hydrothermal synthesis, probe and bath sonication	[74]
All‐inorganic inks	Powders of Cu and Se	Cu_2_Se	/	Se ion binder	Glycerol	Ball milling and centrifugally mixing	[31]
	Granules of Bi, Sb, Te, and Se	Bi_0.55_Sb_1.45_Te_3_ and Bi_2_Te_2.7_Se_0.3_	/	Sb_2_Te_4_ ChaM	Glycerol	Ball milling and centrifugally mixing	[75]
	Powders of Pb, Te, Sb, and Na	Pb_1‐x_Na_x_Te and Pb_1‐x_Sb_x_Te	/	Te powder	Glycerol	Ball milling under a N_2_ atmosphere and centrifugally mixing	[39]
	Granules of Bi, Sb, and Te	Bi_x_Sb_2−x_Te_3_	/	Sb_2_Te_4_ ChaM	Glycerol	Ball milling and centrifugally mixing	[76]
	Particles of Bi, Sb, and Te	Bi_0.55_Sb_1.45_Te_3_	/	/	Glycerol	Ball milling in an Ar atmosphere, ultrasonication, and stirring	[34]
	TeO_2_ and Bi(NO_3_)_3_·5H_2_O	Bi_2_Te_3_ nanowires	/	/	DMSO	Hydrothermal synthesis at 160 °C, centrifugating at RT, and grinding	[94]
	/	Ag_2_Se particles	/	/	Glycerol	Stirring	[42]
	Elemental powders of Bi, Sb, Te, Se, and Pb	Pb_x_(Bi_0.5_Sb_1.5_)_1−x_Te_3_ and Bi_2_Te_2.73_Se_0.3_	BCS and DBP	PLG	DCM	Melting at 800 °C under vacuum and ball milling	[84]
	/	Bi_0.4_Sb_1.6_Te_3_ and carbon black	/	PLA	CLM	Stirring	[79]
Organic/inorganic hybrid inks	/	Bi_0.4_Sb_1.6_Te_3_ and Ag	/	PLA	CLM	Stirring	[80]
	/	Bi_0.5_Sb_1.5_Te_3_ and Bi_2_Te_3_	/	PVP	DMF	Stirring	[81]
	/	PEDOT:PSS and MoS_2_	/	/	Ethyl alcohol	Stirring	[32]
	/	Bi_0.5_Sb_1.5_Te_3.0_ and Bi_2.0_Te_2.7_Se_0.3_	Te and Bi particles	PAA and PEI	Deionized water	Ball milling under an inert atmosphere	[37]
	Elemental powders of Nb, Co, and Sb	Nb_1‐x_CoSb	DBP and EGBE	PS	DCM	Blending under an Ar atmosphere, cold pressing, annealing at 1273 K, ball milling under an Ar atmosphere, and evaporating at 333 K	[85]
	/	Bi_0.5_Sb_1.5_Te_3.0_ and Bi_2.0_Te_2.7_Se_0.3_	PAA and PEI	MC	Deionized water	Ball milling under an inert atmosphere	[87]
	Granules of Bi, Sb, and Te	Bi_0.5_Sb_1.5_Te_3_	/	Pluronic F127	Deionized water	Annealing at 850 °C, ball milling, and stirring	[82]
	Elemental powders of Bi, Sb, and Te	Bi_0.4_Sb_1.6_Te_3_ and Bi_2_Te_2.6_Se_0.4_	/	PVP	DMF	Ball milling under an Ar atmosphere and stirring	[83]
	Elemental powders of Ti, Ni, and Sn	TiNiSn	EGBE and DBP	PS	DCM	Mixing	[86]
	/	Black phosphorus and DMSO‐added PEDOT:PSS	/	/	/	Stirring	[78]
	Elemental powders of Bi, Sb, and Te	Bi_0.5_Sb_1.5_Te_3_	Xanthan gum	Bi nanoparticles and Sb_2_Te_4_ ChaM	EG	High‐energy ball milling	[42]
	/	Carbon nanofibers	NaCl	TPU	Ethanol	Centrifugally mixing	[43]
Carbon nano‐material based inks	/	PAA or PEI doped SWCNTs	/	/	DEG	Ball milling	[33, 35, 41]
	PEDOT:PSS and MWCNTs	PEDOT:PSS/MWCNT hydrogel	/	/	Deionized water	One‐pot hydrothermal method at 90 °C, vacuum freeze‐drying at −65 °C, and ultrasonication	[95]
	/	PEDOT:PSS‐ or PEI‐SWCNTs	Epoxy resin	/	/	Shear mixing	[36]

: PEDOT:PSS = poly(3,4‐ethylenedioxythiophene):poly(styrenesulfonate), DMSO = dimethyl sulfoxide, Triton^TM^ X‐100 = t‐octylphenoxypolyethoxyethanol, PLA = polylactic acid, SAP = superabsorbent‐polymer, Li salt = bis(trifluoromethane)sulfonimide lithium salt, GOPS = (3‐glycidyloxypropyl)trimethoxysilane, PEO = poly(ethylene oxide), Bi = bismuth, Sb = antimony, Te = tellurium, Se = selenium, ChaM = chalcogenidometallate, N_2_ = nitrogen, SbCl_3_ = antimony trichloride, SeO_2_ = selenium dioxide, Sb_2_Te_3_ = antimony telluride, EG = ethylene glycol, Cu = copper, Cu_2_Se = cuprous selenide, Pb = lead, Na = sodium, Bi(NO_3_)_3_·5H_2_O = bismuth nitrate pentahydrate, Bi_2_Te_3_ = bismuth telluride, Ag = silver, BCS = 2‐butoxy ethanol, DBP = dibutyl phthalate, PLG = polylactide‐co‐glycolide, DCM = dichloromethane, CLM = chloroform, PVP = polyvinyl pyrrolidone, DMF = dimethyl formamide, MoS_2_ = molybdenum disulfide, PAA = poly(acrylic acid), PEI = poly(ethylenimine), Nb = niobium, Co = cobalt, EGBE = ethylene glycol butyl ether, PS = polystyrene, MC = methylcellulose, Pluronic F127 = PEO‐PPO‐PEO copolymer, Ti = titanium, Ni = nickel, Sn = tin, NaCl = sodium chloride, TPU = thermoplastic polyurethane, SWCNTs = single‐walled carbon nanotubes, DEG = diethylene glycol, and MWCNTs = multi‐walled carbon nanotubes. ‘/’ represents the absence of certain substance or preparation conditions that are not explicitly stated in the literature. RT stands for room temperature.

## 4 Thermoelectric Materials and Devices Prepared via Solution 3D Printing

### 4.1 Thermoelectric Materials

#### 4.1.1 Flexible Thermoelectric Films

**Figure 3:**
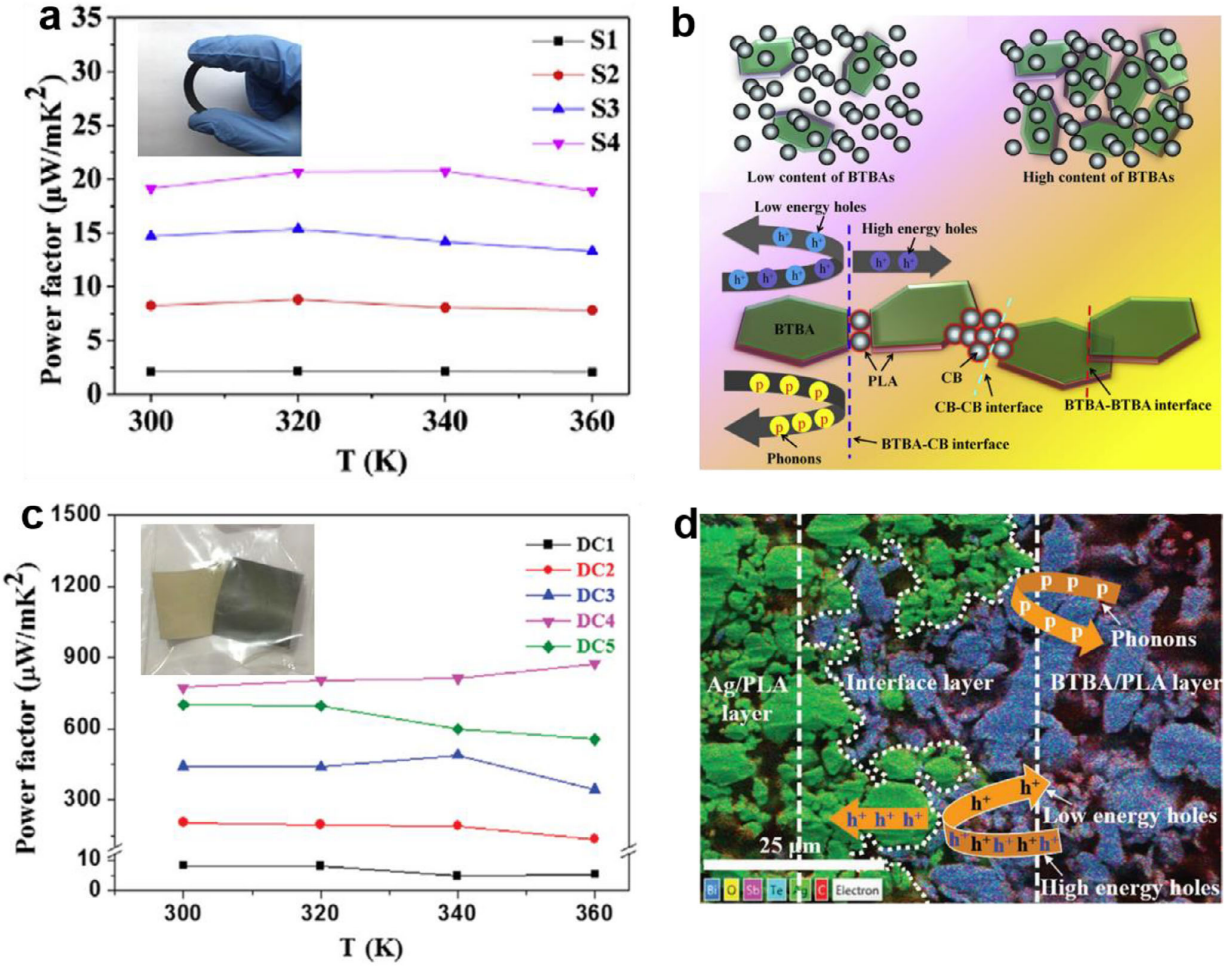
Solution 3D printed flexible films employing insulating binder and mechanisms for thermoelectric performance enhancement. a) Dependence of the *PF* of flexible self‐supporting carbon black/Bi_2_Te_3_ based alloy/polylactic acid (CB/BTBA/PLA) series on test temperature, and b) schematic illustration of phonons and carriers transport across the interfaces in CB/BTBA/PLA composites. Reproduced with permission.^[^ ^79^ ^]^ Copyright 2020, Elsevier. c) Dependence of the *PF* of Bi_2_Te_3_‐based alloy/polylactic‐acid‐silver/polylactic‐acid (BTBA/PLA‐Ag/PLA) double‐layer series on test temperature, and d) EDS layered image for BTBA/PLA‐Ag/PLA with 45.6 vol% Ag in in the Ag/PLA layer (DC5). Reproduced with permission.^[^ ^80^ ^]^ Copyright 2020, Wiley‐VCH.

Solution 3D‐printed thermoelectric films can be fabricated either on flexible substrates^[^ ^32^ ^]^ or as self‐supporting structures via post‐treatments such as drying,^[^ ^79^ ^]^ or soaking in deionized water^[^ ^65^ ^]^ or methanol.^[^ ^78^ ^]^ When printed on thin flexible substrates or containing polymeric components, these composite films generally exhibit good mechanical flexibility, though often at the expense of thermoelectric performance. In recent years, Du et al.^[^ ^32^, ^65^, ^78^, ^79^, ^80^ ^]^ have developed a series of flexible composite thermoelectric films using solution 3D printing, as illustrated in **Figure** **3**.

The first type of composite films employed insulating PLA as the binder in the printing slurries. In 2020, Du et al. fabricated p‐type composite films composed of carbon black (CB), Bi_2_Te_3_‐based alloy (BTBA), and PLA (CB/BTBA/PLA), as shown in Figure 3a.^[^ ^79^ ^]^ At 300 K, increasing the Bi_0.4_Sb_1.6_Te_3_ content from 38.5% to 71.4% (S1–S4) enhanced the *σ* from 5.8 to 13.3 S cm^−1^ and the *S* from 60.2 to 119.9 µV K^−1^. Consequently, the *ZT* value increased from 0.004 to 0.023, with a slight rise in *κ* from 0.15 to 0.25 W m^−1^ K^−1^. As illustrated in Figure 3b, higher BTBA content introduced additional interfaces (BTBA‐BTBA, BTBA‐PLA, CB‐CB, and BTBA‐CB), which promoted hole scattering and contributed to the increase in *S*. A maximum *ZT* of 0.024 was achieved at 320 K with 71.4% Bi_0.4_Sb_1.6_Te_3_ (S4 in Figure 3a). Building on this, Du et al. ^[^ ^80^ ^]^ developed flexible double‐layer composite films (DC1‐DC5) consisting of BTBA/PLA and silver/PLA (BTBA/PLA–Ag/PLA), as shown in Figure 3c. Increasing the Ag content in the Ag/PLA layer from 26.3 to 45.6 vol% significantly enhanced *σ* from 12 to 1710.2 S cm^−1^ while maintaining *S* at ≈80 µV K^−1^ at 300 K. The double‐layer structure facilitated enhanced carrier transport and selective hole scattering at the interface (Figure 3d), resulting in much higher *σ* and *S* than those of single‐layer BTBA/PLA composites. At 360 K, the maximum *PF* reached 875 µW m^−1^ K^−2^ with 41.7 vol% Ag (DC4 in Figure 3c).

**Figure 4:**
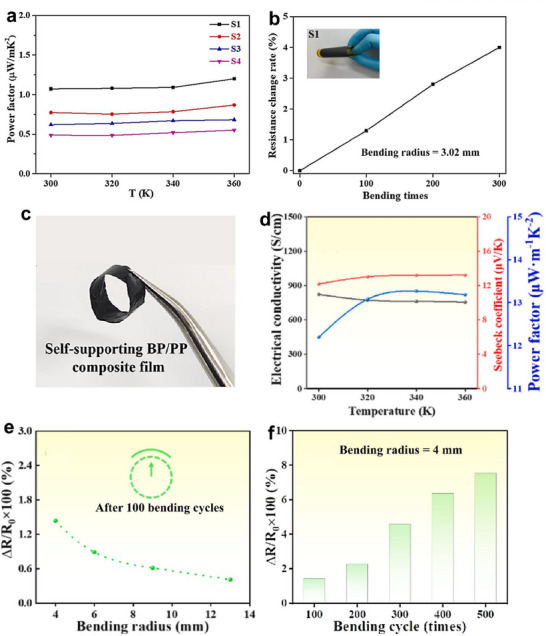
Solution 3D printed flexible films based on conductive PEDOT:PSS slurries and corresponding thermoelectric performance and flexibility characterization. a) Dependence of the *PF* of MoS_2_/PEDOT:PSS series on test temperature, and b) dependence of Δ*R*/*R*_0_ of 69 wt%‐MoS_2_/PEDOT:PSS composite film (S1) on bending cycles. Reproduced with permission.^[^ ^32^ ^]^ Copyright 2021, Elsevier. c) Flexibility demonstration, d) dependence of thermoelectric properties on test temperature, e,f) dependence of Δ*R*/*R*_0_ of 2 wt%‐black phosphorus/PEDOT:PSS (BP/PP) composite film on bending radius and cycles. Reproduced with permission.^[^ ^78^ ^]^ Copyright 2024, Elsevier.

The second type of composite films was based on conductive PEDOT:PSS slurries. As shown in **Figure** **4**a, MoS_2_/PEDOT:PSS composite films were studied by Xu et al.^[^ ^32^ ^]^ Increasing the MoS_2_ content from 69 to 90 wt% (S1–S4) led to a decrease in *σ* from 49.01 to 30.15 S cm^−1^, while the *S* remained relatively stable between 12.7 and 14.79 µV K^−1^ at 300 K. At 360 K, the 69 wt%‐MoS_2_/PEDOT:PSS film (S1) exhibited the highest *PF* of 1.2 µW m^−1^ K^−2^ and demonstrated excellent flexibility, with only a 4% change in resistance after 300 bending cycles at a bending radius of 3.02 mm (Figure 4b). In a related study, all‐organic polyvinyl alcohol (PVA)/PEDOT:PSS composite films were fabricated and evaluated.^[^ ^65^ ^]^ At 300 K, successive treatments with deionized water and DMSO removed excess PVA and PSS, significantly increasing *σ* from 96 to 1085 S cm^−1^, while *S* remained relatively stable (18–20 µV K^−1^). The deionized water‐then‐DMSO‐treated film achieved the highest *PF* of 45 µW m^−1^ K^−2^ and exhibited outstanding mechanical durability, showing only a 1.7% change in resistance after 300 bending cycles. More recently, Du et al. ^[^ ^78^ ^]^ developed self‐supporting black phosphorus/PEDOT:PSS (BP/PP) composite films (Figure 4c). The 2 wt%‐BP/PP film demonstrated the highest *PF* of 13.18 µW m^−1^ K^−2^ at 360 K, with an *σ* of 755.53 S cm^−1^ and a *S* of 13.21 µV K^−1^ (Figure 4d).

To assess the flexibility of these thermoelectric composite films for potential use in wearable electronics, the relative change in electrical resistance (Δ*R*/*R*_0_) is typically measured under varying bending radii and after repeated bending cycles at a fixed, small radius. As shown in Figure 4e, the Δ*R*/*R*_0_ of the solution 3D printed 2 wt% BP/PP composite film increased as the bending radius decreased. After 500 bending cycles at a bending radius of 4 mm, the resistance increased by only 7.5% (Figure 4f), demonstrating good mechanical stability under repetitive deformation.

#### 4.1.2 Rigid Thermoelectric Materials

**Figure 5:**
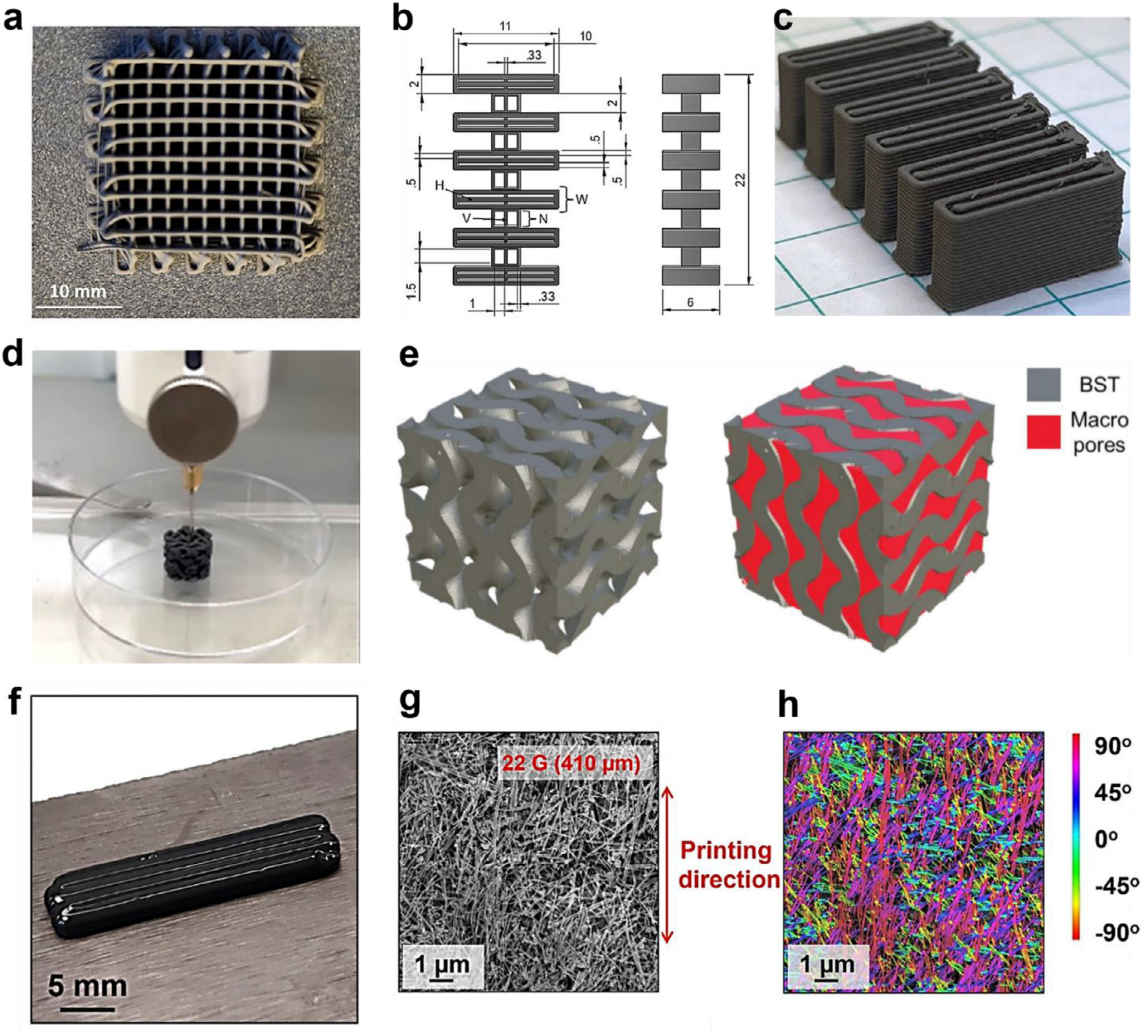
Solution 3D printed non‐flexible thermoelectric materials. a) As‐printed lattice of half‐Heusler alloy Nb_1−_ *_x_* CoSb. Reproduced with permission.^[^ ^85^ ^]^ Copyright 2022, Elsevier. b) Geometry of layered thermoelectric leg where measurements are in mm, and c) as‐printed 3D architectured shape containing Ni and Ti powders. Reproduced with permission.^[^ ^86^ ^]^ Copyright 2023, Elsevier. d) Porous Bi_0.5_Sb_1.5_Te_3_/Pluronic F127 3D structure, and e) its stereolithography model for the macro porosity determining. Reproduced with permission.^[^ ^82^ ^]^ Copyright 2022, American Chemical Society. f) Digital photograph, g) SEM image of nanowire alignment, and h) color‐coded output of the SEM image showing the degree of BTNW alignment of 3D printed Bi_2_Te_3_ nanowires (BTNW) structure. Reproduced with permission.^[^ ^94^ ^]^ Copyright 2023, Royal Society of Chemistry.

Solution 3D printing also enables the fabrication of thermoelectric structures with complex architectures, such as lattice,^[^ ^85^ ^]^ layered,^[^ ^86^ ^]^ and porous^[^ ^82^ ^]^ thermoelectric legs with high specific surface areas, as illustrated in **Figure** **5**. Malki et al.^[^ ^85^ ^]^ printed lattice structures with ≈600 µm diameter struts using an ink composed of pre‐alloyed Nb_1−_ *_x_* CoSb half‐Heusler powder (Figure 5a), achieving a relative density of ≈70%–80% after debinding and vacuum sintering. Based on millimeter‐scale geometric design (Figure 5b), Pröschel et al.^[^ ^86^ ^]^ fabricated layered thermoelectric legs (Figure 5c) from a composite slurry containing Ni and Ti powders. Zhang et al.^[^ ^82^ ^]^ proposed porous Bi_0.5_Sb_1.5_Te_3_/Pluronic F127 gyroid structures (Figure 5d) for waste‐heat energy harvesting via heat pipes. Utilizing a stereolithography (STL) model, the structure achieved a porosity of ≈0.435 (Figure 5e), facilitating efficient heat transfer with minimal impact on fluid flow. Additionally, 3D‐printed Bi_2_Te_3_ nanowire (BTNW) microstructures were fabricated via extrusion along capillary lengths (Figure 5f).^[^ ^94^ ^]^ Scanning electron microscopy (SEM) imaging and corresponding color‐coded mapping confirmed the highly unidirectional alignment of the BTNWs (Figure 5g,h). These examples highlight the architectural versatility of solution 3D printing in producing diverse and structurally sophisticated thermoelectric components, capabilities that remain unattainable with most conventional thermoelectric manufacturing techniques due to the brittleness of inorganic materials.

### 4.2 TEDs

#### 4.2.1 Flexible in‐Plane TEDs With Supporting Base

For in‐plane TEDs fabricated via solution 3D printing, flexible insulating substrates such as Kapton, polyethylene naphthalate (PEN), polyethylene terephthalate (PET), elastomers, polyimide (PI), and polydimethylsiloxane (PDMS) are commonly used.^[^ ^45^ ^]^ Two main assembly strategies are employed: 1) All‐printed TEDs, in which both the thermoelectric legs and conductive interconnects are directly printed onto the flexible substrate; and 2) Hybrid‐assembled TEDs, where thermoelectric legs are printed separately and subsequently bonded to conductive leads. Over the years, a range of flexible in‐plane TEDs based on solution 3D‐printed films, threads, and filaments have been developed, demonstrating the versatility of this technique for constructing advanced, flexible thermoelectric systems.

**Figure 6:**
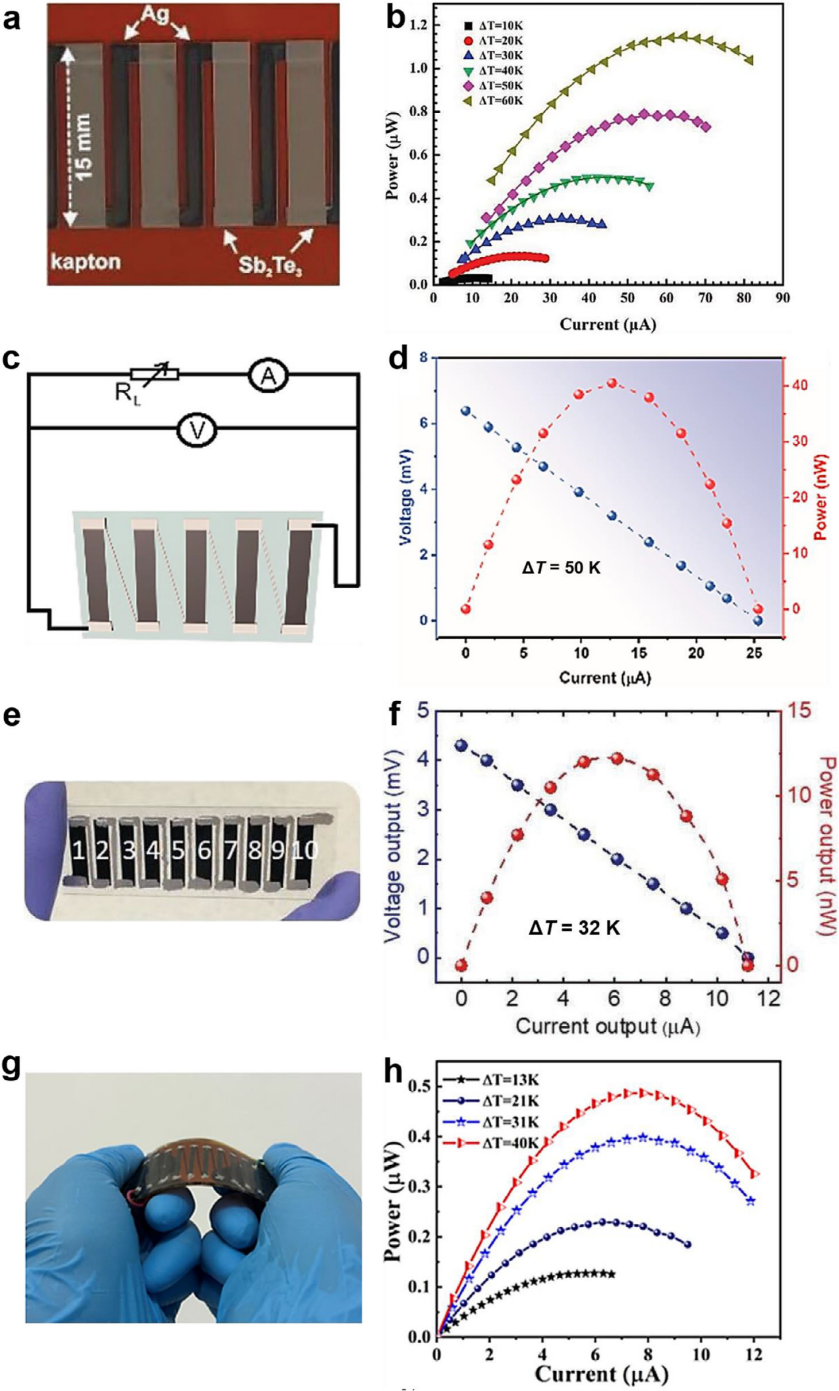
Flexible in‐plane TEGs based on solution 3D printed films and corresponding output performance. a) All‐printed in‐plane TEG based on Sb_2_Te_3_‐Te film and b) *P*_output_ versus *I* at different temperature differences. Reproduced with permission.^[^ ^74^ ^]^ Copyright 2019, Wiley‐VCH. c) All‐printed in‐plane TEG based on PEDOT:PSS/MWCNT film and d) *V*_output_ and *P*_output_ versus *I* at a Δ*T* of 50 K. Reproduced with permission.^[^ ^95^ ^]^ Copyright 2023, MDPI. e) Hybrid‐assembled in‐plane TEG based on PEDOT:PSS/Triton X‐100 film and f) *V*_output_ and *P*_output_ versus *I* at a Δ*T* of 32 K. Reproduced with permission.^[^ ^38^ ^]^ Copyright 2019, Wiley‐VCH. g) Hybrid‐assembled in‐plane TEG based on Bi_0.55_Sb_1.45_Te_3_ film and (h) *P*_output_ versus *I* at different temperature differences. Reproduced with permission.^[^ ^34^ ^]^ Copyright 2024, American Chemical Society.

Thanks to the precise dimensional control enabled by solution 3D printing, all‐printed in‐plane TEDs exhibit highly uniform geometries. Dun et al.^[^ ^74^ ^]^ synthesized Sb_2_Te_3_ ink with an additional Te secondary phase, yielding Sb_2_Te_3_–Te films with an *σ* of 560 S cm^−1^, a *S* of 198 µV K^−1^, and a resultant high *PF* of 22 µW cm^−1^ K^−2^ at 496 K. A planar, all‐printed TEG with four legs (**Figure** **6**a) produced a *P*_max_ of 1.1 × 10^−3^ mW at a Δ*T* of 60 K (Figure 6b), corresponding to a maximum output power density (*P*_dmax_) of 7.65 mW m^−2^ along the in‐plane direction. Similarly, Li et al.^[^ ^95^ ^]^ developed PEDOT:PSS/MWCNT hydrogels via hydrothermal synthesis and printed films from aqueous dispersions containing 2 wt% of freeze‐dried PEDOT:PSS/MWCNT aerogel. As shown in Figure 6c, a TEG composed of five *p*‐type PEDOT:PSS/MWCNT legs connected in series with silver leads was directly printed, achieving a *P*_max_ of 40.48 × 10^−6^ mW at Δ*T* = 60 K.

In hybrid‐assembled in‐plane TEDs, the thermoelectric legs are first printed, followed by the application of silver paste or metallic wires (e.g., copper) as interconnects between the legs. Kee et al.^[^ ^38^ ^]^ fabricated a flexible planar TEG by directly printing PEDOT:PSS/Triton X‐100 films as thermoelectric legs and using silver paste as inter‐electrodes. The 10‐leg TEG (Figure 6e) generated a *V*_oc_ of 4.3 mV at a Δ*T* of 32 K, with a *P*_max_ of 1.2 × 10^−2^ mW and a corresponding *P*_dmax_ of 68 mW m^−2^ (Figure 6f). Cui et al.^[^ ^34^ ^]^ developed a 10‐leg in‐plane TEG using solution 3D printed p‐type Bi_0.55_Sb_1.45_Te_3_ films, with copper wires attached via silver paste (Figure 6g). The printed Bi_0.55_Sb_1.45_Te_3_ film exhibited an excellent *S* of 288 µV K^−1^ at 300 K. The assembled TEG achieved a *P*_max_ of 4.9 × 10^−4^ mW under a Δ*T* of 40 K (Figure 6h).

**Figure 7:**
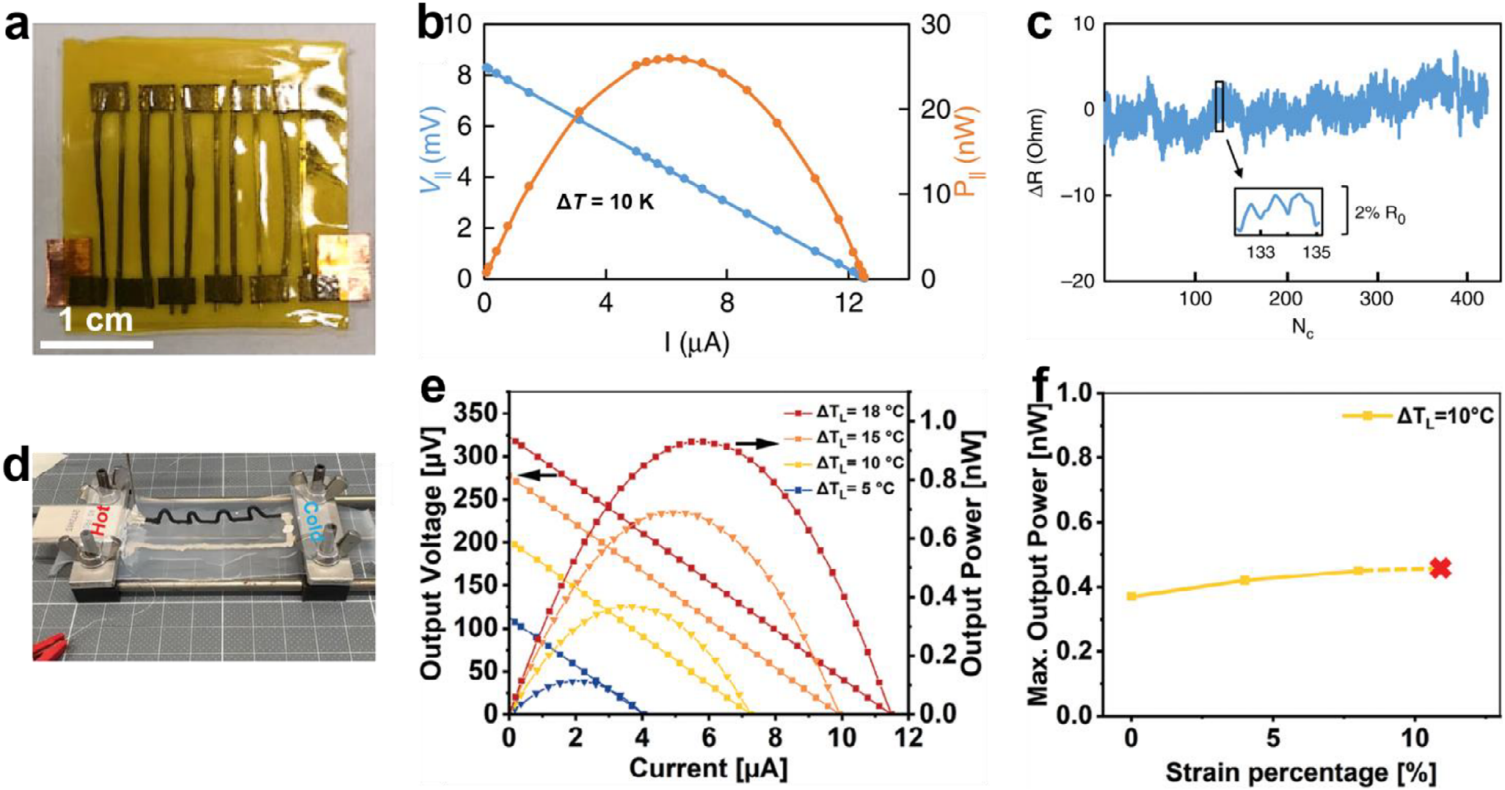
Flexible in‐plane TEGs based on solution 3D printed threads/lines and corresponding output performance and flexibility characterization. a) Hybrid‐assembled in‐plane TEG based on Pb*_x_* (Bi_0.5_Sb_1.5_)_1−_ *_x_* Te_3_ thread with *x* = 0.0025 and Bi_2_Te_2.73_Se_0.3_ thread, b) *V*_output_ and *P*_output_ versus *I* at a Δ*T* of 10 K, and c) bending studies of Pb*_x_* (Bi_0.5_Sb_1.5_)_1−_ *_x_* Te_3_ thread indexed by cycle numbers with a bending radius of 6 mm. Reproduced with permission.^[^ ^84^ ^]^ Copyright 2019, Springer Nature. d) Hybrid‐assembled in‐plane TEG based on GOPS/Li salt/PEDOT:PSS line, e) *V*_output_ and *P*_output_ versus *I* at different temperature differences, and f) *P*_max_ versus applied strain with a Δ*T* of 10 °C across the device. Reproduced with permission.^[^ ^93^ ^]^ Copyright 2025, Wiley‐VCH.

3D‐printed flexible threads and stretchable lines can also serve as thermoelectric legs, addressing the ongoing challenge of developing mechanically compliant power sources for flexible TEGs. Peng et al.^[^ ^84^ ^]^ fabricated continuous, flexible thermoelectric threads via solution extrusion of Bi_2_Te_3_‐based composite inks. In a flexible planar TEG configuration (**Figure** **7**a), *p*‐type Pb*_x_* (Bi_0.5_Sb_1.5_)_1−_ *_x_* Te_3_ (*x* = 0.0025) and *n*‐type Bi_2_Te_2.73_Se_0.3_ threads were alternately printed on Kapton substrates and connected in series using flexible carbon paper. The 5‐pair TEG produced a *V*_oc_ of 8.5 mV at a Δ*T* of 10 K and a *P*_max_ of 2.6 × 10^−5^ mW (Figure 7b). The *p*‐type threads exhibited less than a 2% increase in resistance after hundreds of bending cycles at a radius of 6 mm (Figure 7c), and the *S* remained stable at 225 ± 12 µV K^−1^ after 400 reciprocal bends, confirming their mechanical reliability.^[^ ^84^ ^]^ Additionally, a stretchable in‐plane TEG based on 3D‐printed out‐of‐plane arches was fabricated (Figure 7d), where alternating p‐n pairs were directly printed onto an elastomer substrate. The *p*‐type ink consisted of PEDOT:PSS mixed with bis(trifluoromethane)sulfonimide lithium salt as a plasticizer and (3‐glycidyloxypropyl)trimethoxysilane as a crosslinker, while stretchable silver paste served as the electrode.^[^ ^93^ ^]^ At a Δ*T* of 18 K, the device generated a *V*_oc_ of 0.32 mV and a *P*_max_ of 9.5 × 10^−7^ mW (Figure 7e). Notably, *P*_max_ slightly increased under applied strain (Figure 7f), highlighting its potential for wearable thermoelectric applications.

**Figure 8:**
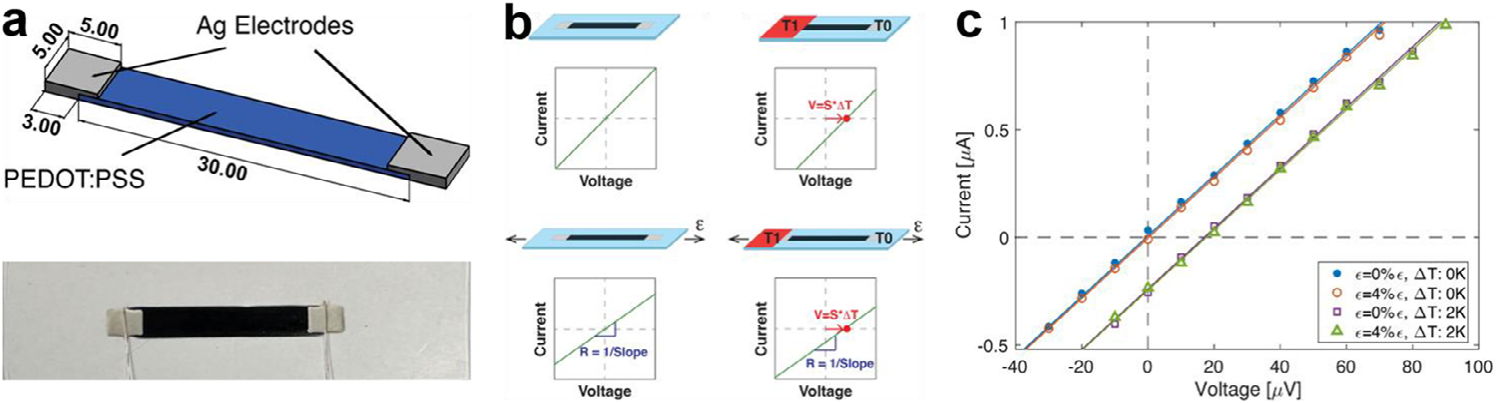
Flexible in‐plane sensor based on solution 3D printed individual thermoelectric leg and corresponding *I‐V* curve responses to strain‐temperature stimulations. a) Strain‐temperature dual‐mode sensor based on 3D printed PEDOT:PSS/polyethylene oxide (PEO) film, b) corresponding *I–V* curve responses during sensing, and c) outline of *I*–*V* curve responses under simultaneous strain and temperature. Reproduced with permission.^[^ ^40^ ^]^ Copyright 2024, IEEE.

In addition to power generation, individual thermoelectric legs based on solution 3D printed flexible films can also function as sensors. As shown in **Figure** **8**a–c, a PEDOT:PSS‐based sensor was fabricated by printing a PEDOT:PSS/poly(ethylene oxide) (PEO) composite ink onto a PDMS substrate.^[^ ^40^ ^]^ The resulting thermoelectric film exhibited a *S* of 10.68 µV K^−1^ at 300 K. The specific sensing applications of this device will be discussed in a later section.

**Figure 9:**
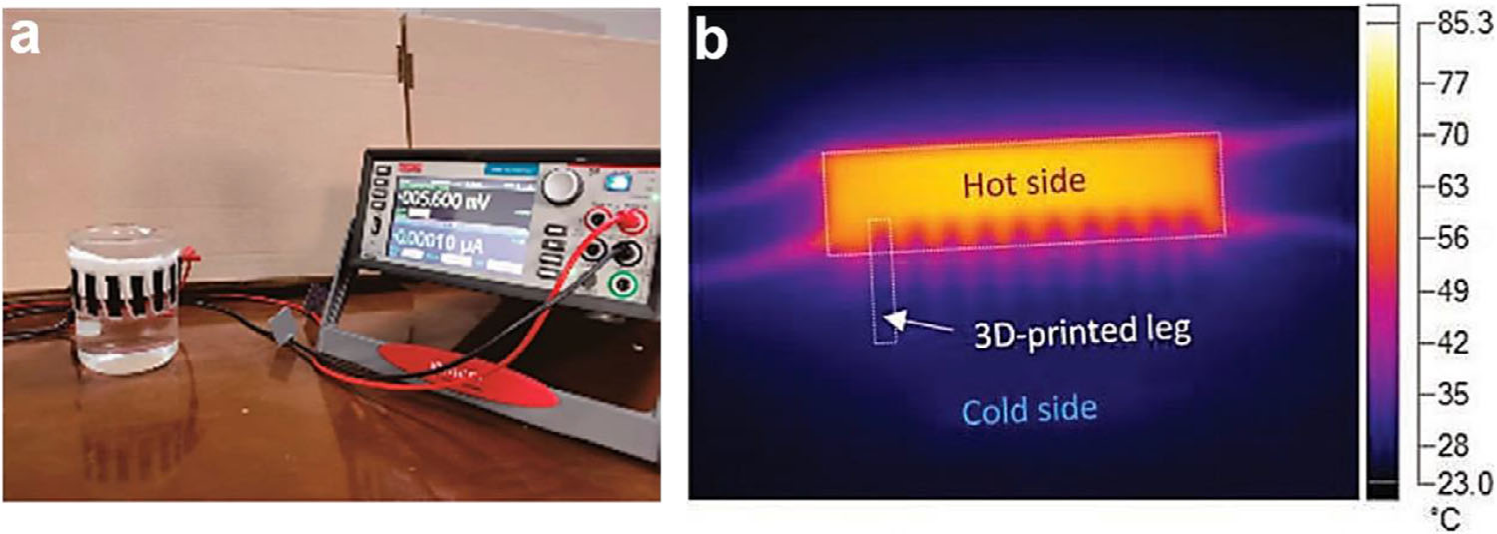
Examples of application scenarios for solution 3D‐printed in‐plane TEDs. a) Photograph of the all‐printed in‐plane TEG based on PEDOT:PSS/MWCNT film, when wrapping around a beaker containing warm water. Reproduced with permission.^[^ ^95^ ^]^ Copyright 2023, MDPI. b) An infrared thermal image of the hybrid‐assembled in‐plane TEG based on PEDOT:PSS/Triton X‐100 film, showing the thermal gradient in the sample. Reproduced with permission.^[^ ^38^ ^]^ Copyright 2019, Wiley‐VCH.

As demonstrated above, solution 3D‐printed in‐plane TEDs can be readily fabricated by printing slurries onto insulating polymer substrates. These devices exhibit excellent flexibility due to the combination of inherently flexible components and substrates. However, practical challenges arise when deploying in‐plane TEDs for large‐area waste heat recovery (e.g., human skin, heating pipelines, etc.). As shown in **Figure** **9**a, when a PEDOT:PSS/MWCNT film‐based TEG was wrapped around a beaker containing warm water,^[^ ^95^ ^]^ one end of the device had to be separated from the beaker wall using an additional thermal‐insulating layer to maintain a continuous temperature gradient. In another scenario (Figure 9b), establishing a temperature difference required individual heat sources at each end of the device: the PEDOT:PSS/Triton X‐100 film‐based TEG generated an in‐plane temperature gradient between one end placed on a Peltier heater and the other exposed to ambient air.^[^ ^38^ ^]^

#### 4.2.2 Flexible Out‐of‐Plane TEDs With Supporting Base

Compared to in‐plane TEDs, flexible out‐of‐plane TEDs offer more efficient heat collection and energy conversion by aligning the heat flow direction parallel to the thermoelectric legs. This configuration allows direct attachment of TEDs to irregular heat source surfaces, significantly enhancing their practicality. In contrast, in‐plane TEGs require the cold ends to be separated from the heat source to maintain a temperature gradient, complicating integration in real‐world scenarios. To fabricate flexible out‐of‐plane TEDs using solution 3D printing, thermoelectric inks are typically deposited directly onto flexible substrates engineered with 3D structures or localized protrusions, promoting effective heat transfer and seamless integration with various surfaces.

**Figure 10:**
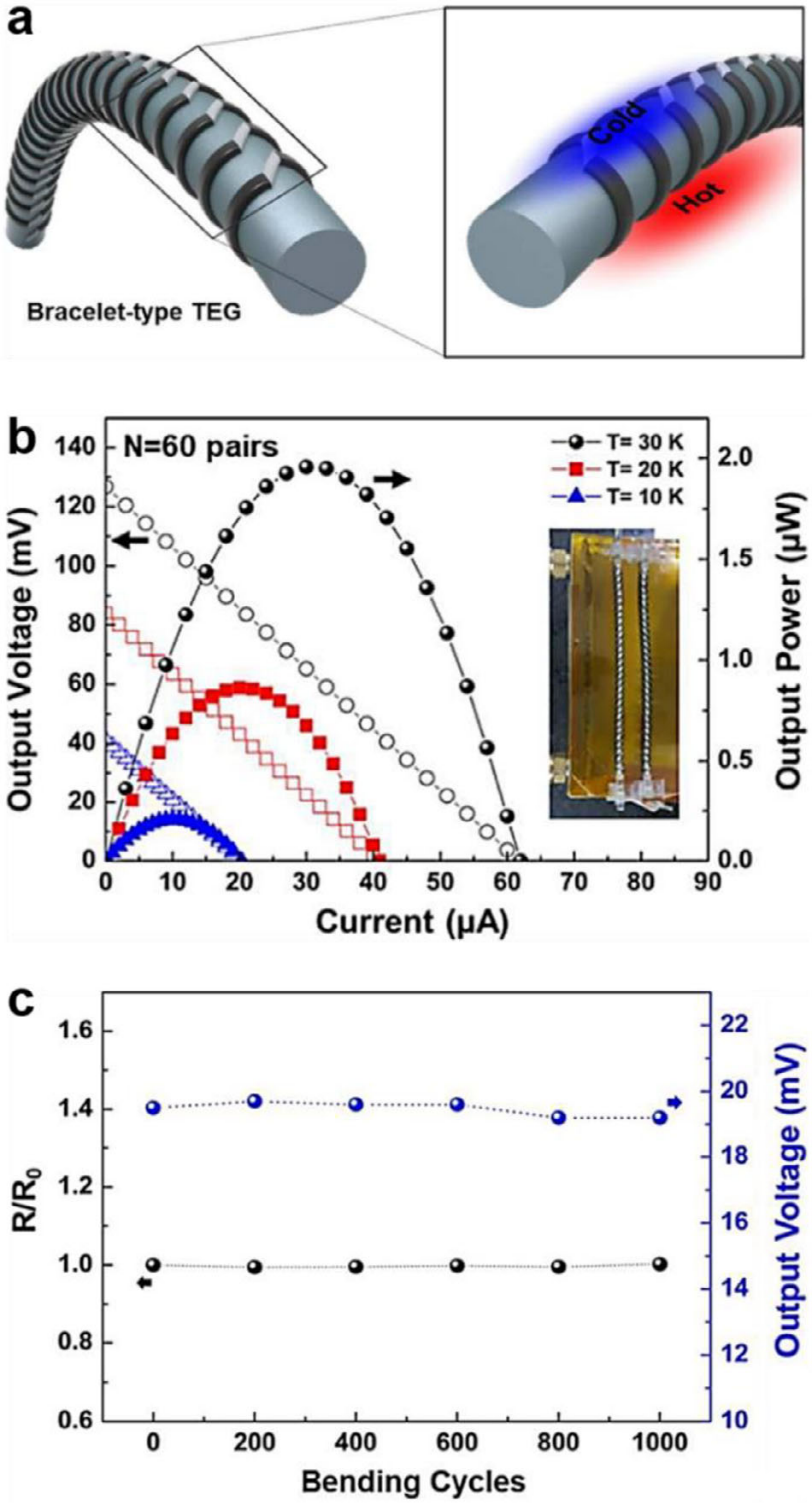
Flexible, directly‐printed bracelet‐type TEG with PAA‐doped and PEI‐doped SWCNTs solutions as *p*‐type and *n*‐type thermoelectric inks. a) Illustration showing the fabrication process and structural diagram of a bracelet‐type TEG by printing inks on a flexible polyurethane (PU) cable, b) *V*_output_ and *P*_output_ versus *I* of the bracelet‐type TEG with 60 p–n pairs, and c) resistance change and *V*_output_ of a bracelet‐type TEG with 30 p–n pairs bent with bending radius of 7.5 cm as a function of bending cycle. Reproduced with permission.^[^ ^35^ ^]^ Copyright 2018, Royal Society of Chemistry.

As shown in **Figure** **10**a, Park et al.^[^ ^35^ ^]^ used PAA‐doped and PEI‐doped SWCNT solutions as *p*‐type and *n*‐type thermoelectric inks, respectively, and directly printed them onto a flexible polyurethane (PU) cable with a 3 mm diameter. Silver inter‐electrodes were also printed to complete the all‐printed bracelet‐type TEG, which contained 60 p–n pairs. This device generated a *V*_oc_ of 130 mV at a Δ*T* of 30 K and a *P*_max_ of 1.95 × 10^−3^ mW (Figure 10b). Additionally, the bracelet‐type TEG exhibited excellent mechanical durability. After 1000 bending cycles at a bending radius of 7.5 cm, the electrical resistance and *V*_output_ remained nearly unchanged (Figure 10c), confirming that no degradation in thermoelectric performance occurred under mechanical stress.

**Figure 11:**
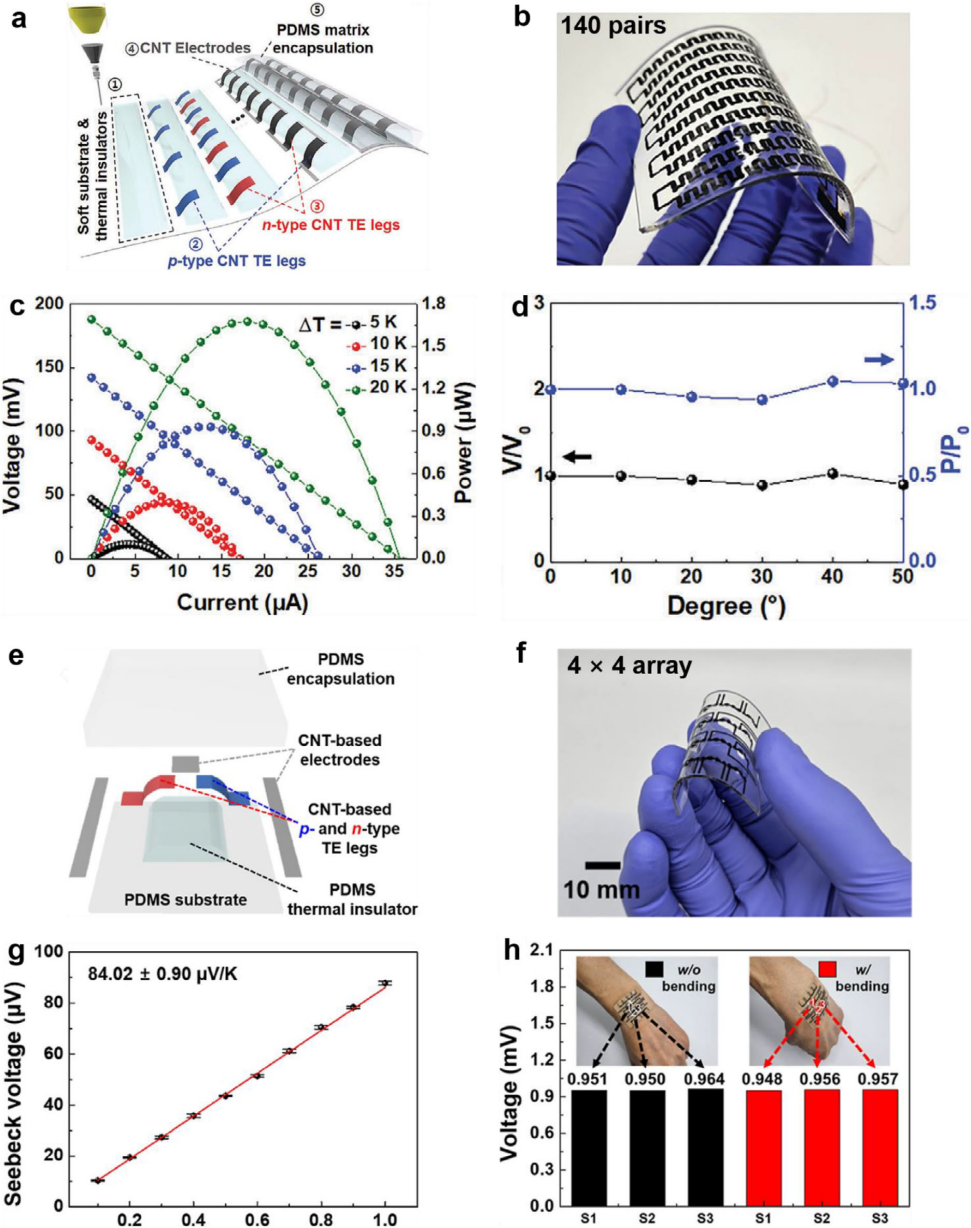
Flexible, directly‐printed 3D‐compliant TEDs with PAA‐doped and PEI‐doped SWCNTs solutions as p‐type and n‐type thermoelectric inks. a) Design concept and the fabrication process of the 3D‐compliant TEGs printed on a flexible polydimethylsiloxane (PDMS) matrix with 1 mm‐thickness thermal insulators, b,c) a 3D‐compliant TEG with 140 p‐n pairs and its corresponding *V*_output_ and *P*_output_, and d) changes in *V*_oc_ and *P*_output_ of a 3D‐compliant TEG with 35 p–n pairs according to deformation at different angles. Reproduced with permission.^[^ ^33^ ^]^ Copyright 2023, Wiley‐VCH. e) Scheme illustration showing the design of a sensing unit for 3D‐printed soft temperature sensors, printed on a PDMS‐based ultrathin substrate and thermal insulators, f,g) the as‐prepared 4 × 4 temperature sensor array and its corresponding Seebeck voltage to temperature gradient from 0.1 to 1 K, and h) Seebeck voltage changes of sensing units during wrist movements ranging from 0° to 60°. Reproduced with permission.^[^ ^41^ ^]^ Copyright 2024, American Chemical Society.

As shown in **Figure** **11**, Hwang et al.^[^ ^33^, ^41^ ^]^ also developed PAA‐ and PEI‐doped SWCNT solutions as p‐type and n‐type thermoelectric inks, respectively, and designed both a 3D‐compliant TEG and a thermoelectric‐powered temperature sensor on soft PDMS substrates featuring protruding thermal insulators. Each *p*–*n* pair was constructed by sequentially printing the *p*‐ and *n*‐type inks along the thermal insulators, with undoped SWCNTs used as interconnects between thermoelectric legs. For the 3D‐compliant TEGs, all p–n pairs were connected in series (Figures 11a). The fully printed 3D‐compliant TEG, comprising 140 p–n pairs, generated a *V*_oc_ of 188.08 mV at a Δ*T* of 20 K, with a *P*_max_ of 1.68 × 10^−3^ mW (Figure 11b,c). Furthermore, the 3D‐compliant TEGs showed excellent conformability and mechanical robustness. As illustrated in Figure 11d, they maintained intimate contact with the human wrist, and their *V*_oc_ and *P*_output_ remained stable even when the wrist was bent to increasingly steep angles. For the soft 3D‐printed temperature sensors, a single p–n pair served as a sensing unit (Figure 11e,f). The *p*‐type and *n*‐type legs exhibited a *S* of 59.19 and −27.59 µV K^−1^ at 300 K, respectively. Thus, the sensors demonstrated a high temperature sensitivity of 84.02 ± 0.90 µV K^−1^ (Figure 11g), enabling rapid mapping of localized thermal distributions. They also possessed superior mechanical reliability. As shown in Figure 11h, the *V*_oc_ of each sensing unit remained nearly constant during wrist bending from 0 to 60°. These demonstrations underscore the design flexibility of solution 3D printing and highlight its potential for scalable fabrication of efficient 3D TEDs for self‐powered, flexible, and wearable applications.

#### 4.2.3 Flexible Free‐Standing Out‐of‐Plane TEDs

Free‐standing out‐of‐plane TEDs are constructed by connecting 3D‐printed thermoelectric blocks with integrated electrodes and conductive leads. Unlike the supported out‐of‐plane TEDs discussed in **Section** **4.2.2**, the thermoelectric legs in free‐standing configurations are typically cuboidal and function independently without requiring a flexible substrate. This design facilitates more direct and convenient utilization of out‐of‐plane temperature gradients.

**Figure 12:**
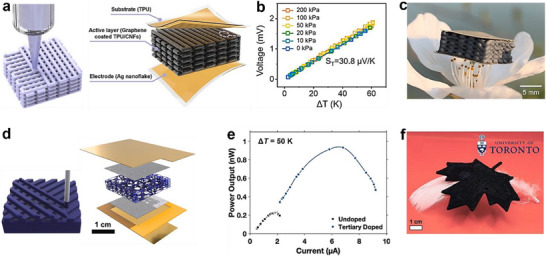
Flexible free‐standing out‐of‐plane TEDs based on individual thermoelectric modules. a) The overall assembly of an all‐in‐one flexible pressure‐temperature sensor based on thermoplastic polyurethane/carbon nanofiber (TPU/CNFs) sponge, b) the Seebeck coefficient of the sensor at different applied force, and c) the sensor on the stamens of a pear blossom showing its light weight. Reproduced with permission.^[^ ^43^ ^]^ Copyright 2021, Elsevier. d) Exploded view of the assembled TE module based on PEDOT:PSS/SAP aerogel, e) *P*_output_ versus *I* at a Δ*T* of 50 K, and f) corresponding aerogel maple‐leaf resting on a piece of feather. Reproduced with permission.^[^ ^30^ ^]^ Copyright 2023, Elsevier.

As shown in **Figure** **12**a, Yin et al.^[^ ^43^ ^]^ fabricated a multilayer‐structured sponge using TPU/carbon nanofiber (TPU/CNF) inks and developed an all‐in‐one flexible pressure–temperature sensor based on a graphene‐coated TPU/CNF sponge. Thanks to the 3D‐printed multilayer architecture and argon plasma treatment of the graphene sheets, the sensor exhibited minimal crosstalk between piezoresistive and thermoelectric signals. It achieved high pressure sensitivity (0.14 kPa^−1^ in the 0–60 kPa range) and a temperature sensitivity of 30.8 µV K^−1^ (Figure 12b). Notably, the ultralight sensor was light enough to rest on the stamens of a pear blossom (Figure 12c). Similarly, Li et al.^[^ ^30^ ^]^ developed a 3D thermoelectric module based on a geometry‐controlled PEDOT:PSS/SAP aerogel, fabricated via solution 3D printing combined with tertiary doping (Figure 12d). The aerogel exhibited a *σ* of ≈10 S cm^−1^ and an ultralow *κ* of <0.1 W m^−1^ K^−1^. The corresponding 3D thermoelectric module delivered an *P* of 9.3 × 10^−7^ mW (Figure 12e). The printed maple leaf–shaped aerogel film was light enough to rest on a feather, further emphasizing its ultralight nature (Figure 12f).

**Figure 13:**
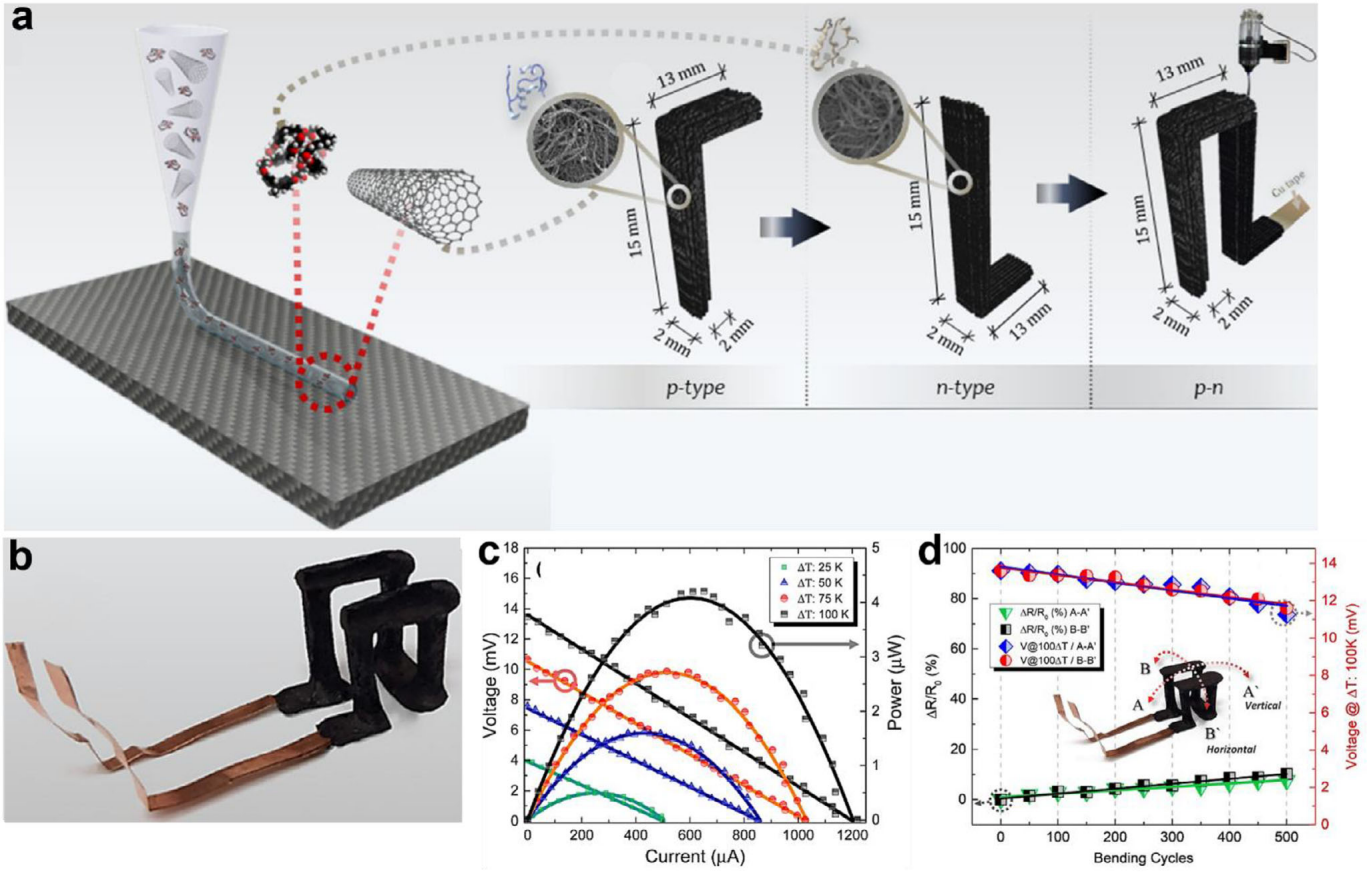
Flexible free‐standing out‐of‐plane TEDs shaped by integrally printing of thermoelectric legs and inter‐electrodes. a) Printing process of PEDOT:PSS‐SWCNTs/epoxy resin block and PEI‐SWCNTs/epoxy resin block based TE materials, b) device architecture of the 3D carbon‐based TEG, c) *V*_output_ and *P*_output_ versus *I* at different temperature differences, and d) Δ*R*/*R*_0_ and *V*_output_ at a Δ*T* of 100 K in various bending cycles. Reproduced with permission.^[^ ^36^ ^]^ Copyright 2024, Royal Society of Chemistry.

In addition to being assembled from individual thermoelectric modules, flexible free‐standing out‐of‐plane TEDs can also be shaped by integrally printing of thermoelectric legs and inter‐electrodes. Mytafides et al. ^[^ ^36^ ^]^ reported a unibody design for a flexible 3D carbon‐based TEG (3D‐CTEG), in which both the thermoelectric legs and inter‐electrodes were fabricated using SWCNT‐based inks (**Figure** **13**a). The printed p‐type and n‐type 3D thermoelements, composed of PEDOT:PSS–SWCNT/epoxy resin and PEI–SWCNT/epoxy resin composites, exhibited *PF*s of 102 and 75 µW m^−1^ K^−2^, respectively. Utilizing undoped SWCNT/epoxy resin as inter‐electrodes, the 3D‐CTEG (Figure 13b) generated a *V*_oc_ of 13.6 mV and a *P*_max_ of 4.1 × 10^−3^ mW at a Δ*T* of 100 K (Figure 13c), the highest reported value for flexible 3D‐printed TEGs to date. Mechanical flexibility tests revealed Δ*R*/*R*_0_ values of 7.6% and 10.3% along the A–A’ and B–B’ axes, respectively, at a bending radius of 2 cm, while the *V*_output_ remained nearly unchanged (Figure 13d). These results exemplify innovative strategies for advancing the integration flexibility of solution‐based 3D printing, enabling the creation of multifunctional materials and next‐generation conceptual devices.

#### 4.2.4 Non‐Flexible Conformal TEDs

**Figure 14:**
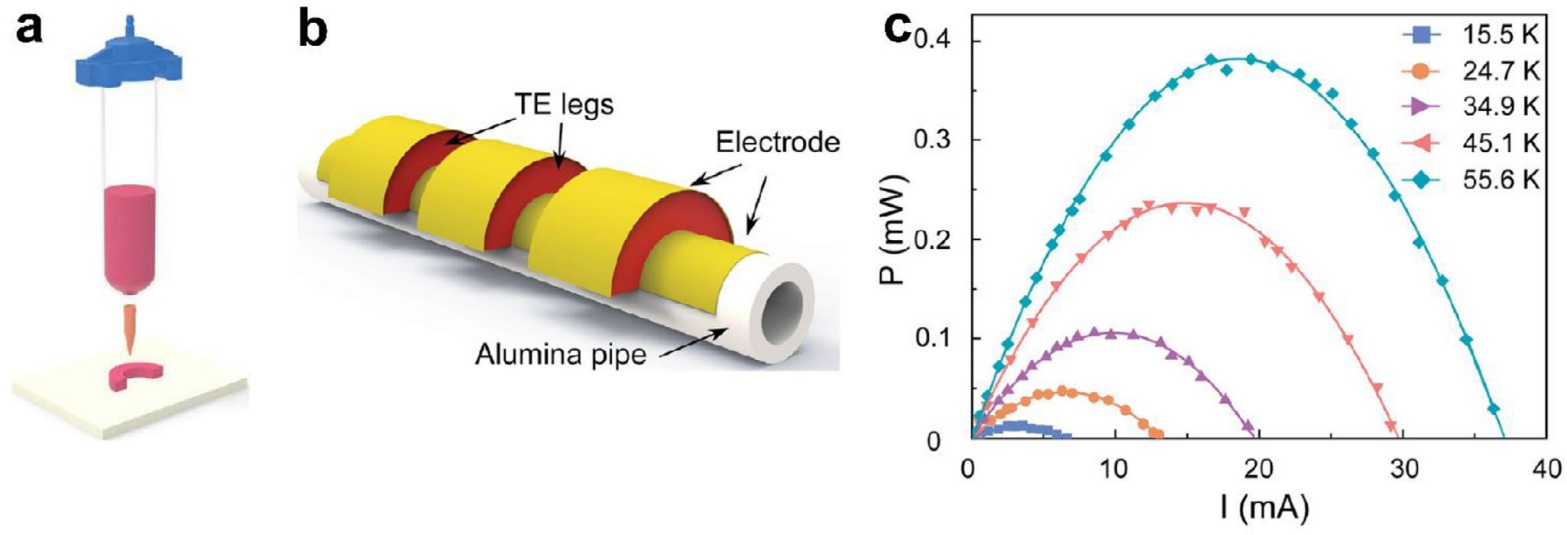
Conformal TEGs with solution 3D printed hemicyclic thermoelectric blocks and corresponding output performance. a) Diagram for solution 3D printing of PAA‐PEI‐Bi_0.5_Sb_1.5_Te_3.0_ and PAA‐PEI‐Bi_2.0_Te_2.7_Se_0.3_ half‐annular rings, b) schematic illustration of the corresponding conformal TEG with 3‐pair p–n half‐annular rings, and c) *P*_output_ versus *I* at different temperature differences. Reproduced with permission.^[^ ^37^ ^]^ Copyright 2022, Royal Society of Chemistry.

Herein, conformal TEDs refer to those incorporating rigid thermoelectric elements, such as hemicyclic or circular blocks, specifically designed and printed to fit curved heat sources of defined dimensions, as shown in **Figure** **14**. Conformal and cylindrical configurations effectively minimize heat loss at the interface between the TED and the heat source, thereby enhancing overall performance. These devices are typically fabricated using all‐inorganic thermoelectric inks or composite inks in which the thermoelectric elements become predominantly inorganic following post‐treatment processes such as annealing.

As illustrated in Figure 14a, Wang et al.^[^ ^37^ ^]^ developed half‐annular rings by solution 3D printing of viscoelastic PAA‐PEI‐Bi_0.5_Sb_1.5_Te_3.0_ and PAA‐PEI‐Bi_2.0_Te_2.7_Se_0.3_ inks, followed by 24 h drying and 2 h annealing (450 °C). The corresponding conformal TEG, with three pairs of p–n half‐annular rings (Figure 14b), delivered a *V*_oc_ of 40.61 mV and a *P*_max_ of 0.38 mW at a Δ*T* of 55.6 K (Figure 14c), surpassing the performance of conventional planar TEGs. Similarly, other conformal TEGs based on solution 3D‐printed hemicyclic thermoelectric blocks were demonstrated, including those fabricated from Bi_2_Te_3_‐based inks (Bi_0.4_Sb_1.6_Te_3_ and Bi_2_Sb_2.7_Se_0.3_) using Sb_2_Te_3_ ChaM ions as inorganic binders,^[^ ^73^ ^]^ as well as PAA‐PEI‐Bi_0.5_Sb_1.5_Te_3.0_/MC and PAA‐PEI‐Bi_2.0_Te_2.7_Se_0.3_/MC formulations.^[^ ^87^ ^]^

**Figure 15:**
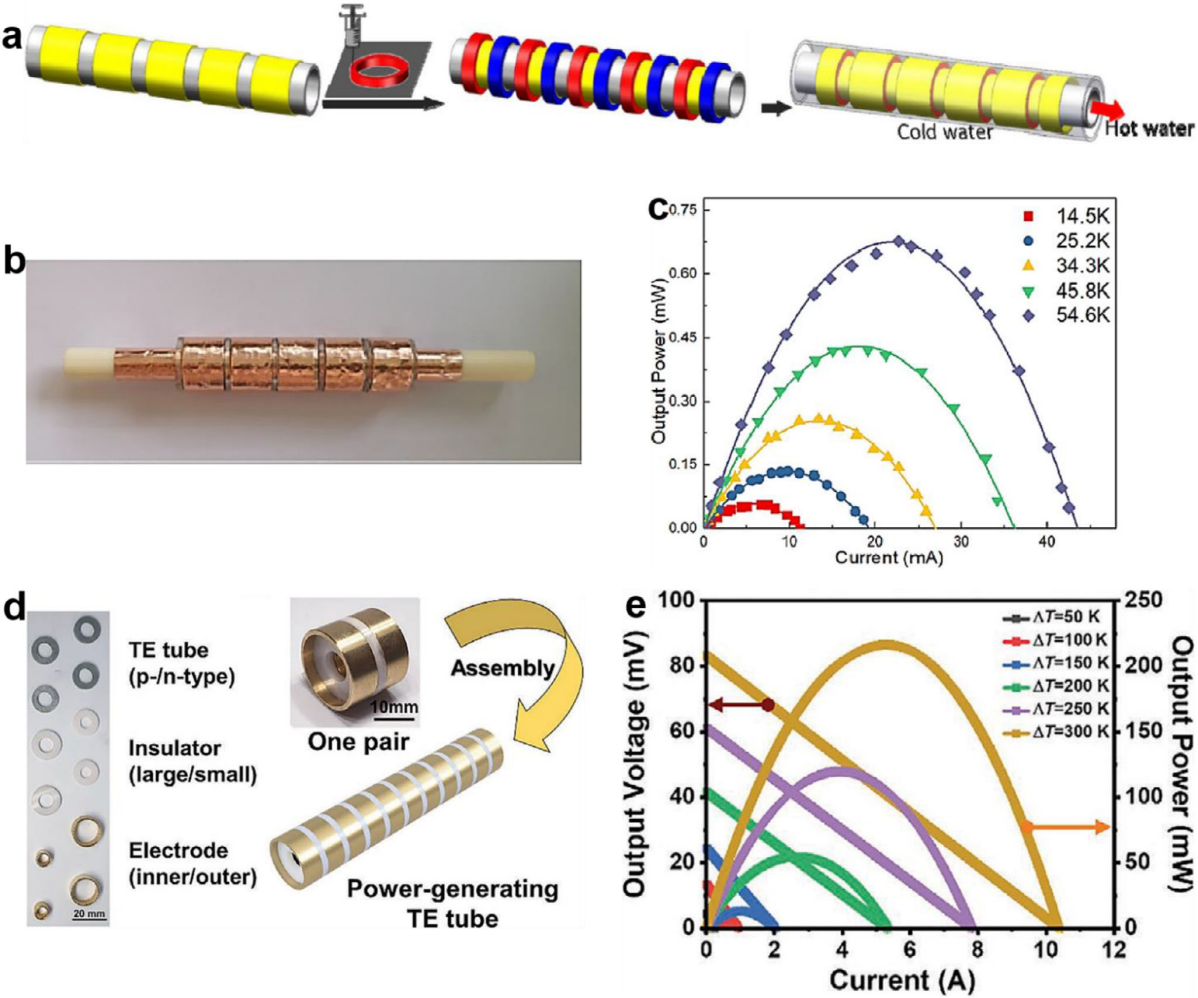
Conformal TEGs with solution 3D printed circular thermoelectric blocks and corresponding output performance. a) Preparation process of the conformal TEG based on Bi_0.5_Sb_1.5_Te_3_/PVP and Bi_2_Te_3_/PVP annular rings, b) photograph of the annular device with five pairs of TE legs, and c) corresponding output power at different temperature differences. Reproduced with permission.^[^ ^81^ ^]^ Copyright 2020, Elsevier. d) Photographs of the unit modules and schematic model of the conformal TEG based on Pb_0.98_Na_0.02_Te and Pb_0.98_Sb_0.02_Te tubes, and e) corresponding output voltage and power at different temperature differences. Reproduced with permission.^[^ ^39^ ^]^ Copyright 2021, Wiley‐VCH.

Additionally, Su et al.^[^ ^81^ ^]^ assembled a conformal TEG using annular rings printed from Bi_0.5_Sb_1.5_Te_3_/PVP and Bi_2_Te_3_/PVP organic–inorganic composite thermoelectric inks (**Figure** **15**a,b). After heat treatment, the printed thermoelectric materials with 91 wt% inorganic filler achieved maximum *ZT* values of 0.104 (*p*‐type) and 0.11 (*n*‐type) at room temperature. The resulting annular TEG, composed of five p–n pairs, delivered a *P*_max_ of 0.68 mW at a Δ*T* of 54.6 K (Figure 15c). Lee et al.^[^ ^39^ ^]^ further advanced this concept by designing a power‐generating thermoelectric tube using printed Pb_0.98_Na_0.02_Te and Pb_0.98_Sb_0.02_Te segments, connected via custom‐designed tubular insulators and electrodes (Figure 15d). With five p–n pairs, the tube produced a *V*_oc_ of 83.2 mV and a *P*_max_ of 216.3 mW at a Δ*T* of 300 K (Figure 15e).^[^ ^39^ ^]^ These developments underscore the potential of solution 3D printing for engineering complex thermoelectric architectures, enabling enhanced energy conversion efficiency for both structural and functional applications.

#### 4.2.5 Others

In addition to the flexible and conformal thermoelectric materials and devices discussed earlier, this section highlights other innovative materials and device architectures fabricated via solution 3D printing. The ink formulations and printed structures presented herein offer valuable insights for further advancing solution 3D printing technology in thermoelectric applications.

**Figure 16:**
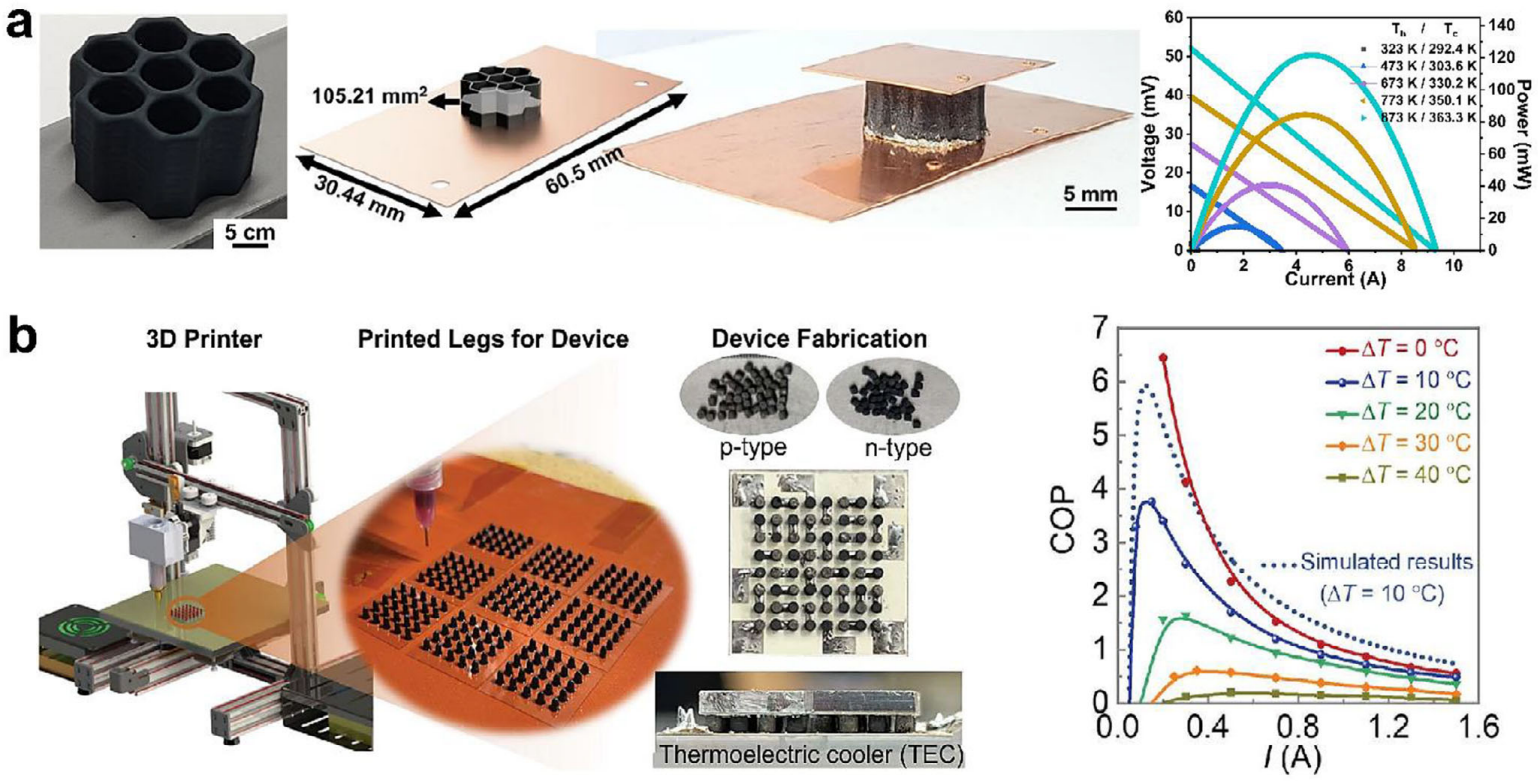
Nonflexible or nonconformal TEDs assembled from solution 3D printed thermoelectric elements and corresponding output performance. a) Cu_2_Se honeycomb‐based TEG. Reproduced with permission.^[^ ^31^ ^]^ Copyright 2021, Springer Nature. b) Printing process of the arrays of TE legs based on (Bi, Sb)_2_Te_3_ and n‐type Ag_2_Se, pictures of the printed TE legs and their assembly into the device, and *COP* of the fabricated TEC as a function of temperature gradient. Reproduced with permission.^[^ ^42^ ^]^ Copyright 2025, American Association for the Advancement of Science.

Choo et al.^[^ ^31^ ^]^ developed an all‐inorganic Cu_2_Se‐based printable ink incorporating Se_8_^2−^ polyanions, which significantly enhanced the ink printability, and proposed a honeycomb‐inspired design for thermoelectric legs. At 1000 K, the printed Cu_2_Se exhibited a *S* of 186 µV K^−1^, a *PF* of 605 µW m^−1^ K^−2^, and a *κ* of 0.5 W m^−1^ K^−1^, resulting in a *ZT* of ≈1.21. As shown in **Figure** **16**a, a single honeycomb‐structured Cu_2_Se leg under a Δ*T* of 509.7 K produced a *P*_max_ of 121 mW, with an *V*_output_ of 52 mV and a *P*_dmax_ of 6.21 × 10^6^ mW m^−2^. In a subsequent study, Choo et al.^[^ ^96^ ^]^ further optimized the geometry of Cu_2_Se‐based thermoelectric legs using 3D printing followed by heat treatment. Compared with the cuboid, truncated pyramid, reverse truncated pyramid, inverse hourglass, multi‐hollow rectangle, Y, and reverse Y shaped design, the hourglass‐shaped design yielded the highest power generation performance among the tested geometries, demonstrating the impact of structural optimization on thermoelectric efficiency.

Segmented thermoelectric leg can also be fabricated via solution 3D printing by sequentially overlaying Bi_0.35_Sb_1.65_Te_3_, Bi_0.5_Sb_1.5_Te_3_, and Bi_0.55_Sb_1.45_Te_3_.^[^ ^76^ ^]^ This compositionally graded structure ensured full compatibility across the operating temperature range and demonstrated a promising strategy for optimizing segmented TEGs. Under a Δ*T* of 236.1 K, the device achieved a *V*_oc_ of 56.7 mV, a *P*_max_ of 260.4 mW, and a *P*_dmax_ of 2.6 × 10^6^ mW m^−2^.

Recently, as shown in Figure 16b, Xu et al.^[^ ^42^ ^]^ fabricated high‐performance 3D‐structured thermoelectric materials using solution 3D printing followed by sintering. The printed materials achieved record‐high *ZT* values at room temperature, 1.42 for *p*‐type (Bi, Sb)_2_Te_3_ and 1.3 for *n*‐type Ag_2_Se. The corresponding TEC exhibited a *COP* of 3.8 under an applied current of 0.15 A, demonstrating a transformative approach to TED fabrication. Kim et al.^[^ ^75^ ^]^ also developed similar micro‐TEG by directly printing vertically aligned thermoelectric filaments from highly viscoelastic inks based on (Bi, Sb)_2_(Te, Se)_3_ particles, eliminating the need for organic binders. These thermoelectric filaments exhibited high *ZT* values of 0.84 (*p*‐type) and 0.37 (*n*‐type) at 300 K, comparable to those of conventional bulk ingots. The resulting micro‐TEG generated substantial temperature gradients, and a configuration of three p–n pairs connected in series achieved a *P*_dmax_ of 4790 mW m^−2^ at a Δ*T* of 82.9 K. These results underscore the significant manufacturing advantages of solution 3D printing over conventional microfabrication methods, which are typically restricted to planar thermoelectric film architectures.

**Table** **3** provides a comprehensive summary of the thermoelectric properties of solution 3D‐printed materials, along with the output performance of their corresponding TEGs under specified operating conditions. Flexible thermoelectric materials and devices are typically fabricated using all‐organic, organic–inorganic hybrid, or carbon nanomaterial‐based inks, with a smaller portion derived from all‐inorganic inks printed on flexible substrates. Due to the flexibility contributed by organic components, post‐processing and device operation must be conducted at relatively low temperatures, which generally results in nanowatt‐level *P*_max_ per degree Kelvin for each p–n pair. In contrast, conformal TEDs are usually fabricated using all‐inorganic inks. As detailed in Table 2, these inks typically consist of inorganic thermoelectric powders dispersed in volatile solvents, sometimes with the addition of ChaM or other inorganic binders. During high‐temperature drying and sintering (e.g., drying at 105 °C for 12 h followed by annealing at 450 °C for 1 h under a nitrogen atmosphere^[^ ^73^ ^]^), the solvents fully evaporate and the ChaM binder fills the voids, leading to densification of the printed structures. As a result, these devices can achieve milliwatt‐level *P*_max_ per degree Kelvin for each p–n pair. Moreover, computer‐aided solution 3D printing enables the fabrication of thermoelectric modules with complex geometries, including filament, cuboid, lattice, and other customized architectures. This design flexibility makes solution 3D printing a highly promising approach for the tailored development of thermoelectric materials and devices with application‐specific shapes and performance requirements.

**Table 3:** Thermoelectric properties of solution 3D printed thermoelectric materials and output performance of their TEGs under corresponding working conditions in the literature.

Material	Type	*T*	*|S|*	*σ*	*PF*	*κ*	*zT*	TEG configuration	Δ*T*	*V*_oc_	*P*_max_	Device dimension	*Pd*_max_	Applicability	Reference
PEDOT:PSS/Triton^TM^ X‐100 film	p	298	14	/	/	/	/	10 legs of 20 mm × 3 mm × 60 µm (L × W × T), in‐plane	32	4.3	1.2 × 10^−2^	/	/	Flexible	[38]
PVA/PEDOT:PSS film	p	300	20	1085	45	/	/	/	/	/	/	/	/	Flexible	[65]
		345	22	1044	50.5	/	/								
PEDOT:PSS/SAP aerogel	p	298	/	10	/	<0.1	/	One single leg with thickness of 3 mm, out‐of‐plane	50	0.69	9.3×10^−7^	/	/	Flexible	[30]
GOPS/Li salt/PEDOT:PSS aerogel filament	p	300	23.5	16	0.88	0.1695±0.0016	1.56 × 10^−3^	One p−n pair, in‐plane	18	0.32	9.5 × 10^−7^	/	/	Flexible	[93]
								One single leg, out‐of‐plane	20	0.275	2 × 10^−7^	/	/	Flexible	[93]
PEDOT:PSS/PEO film	p	300	10.68	/	/	/	/	/	/	/	/	/	/	Flexible	[40]
Sb_2_Te_3_ film	p	300	130	810	1370	/	/	/	/	/	/	/	/	Flexible	[74]
		400	151	680	1550	/	/								
Sb_2_Te_3_‐Te film	p	300	147	630	1360	/	/	4 legs, in‐plane	60	36.6	1.1 × 10^−3^	24 mm × 6 mm (L × T)	7.65	Flexible	[74]
		496	198	560	2200	/	/								
Bi_0.55_Sb_1.45_Te_3_ film	p	300	288	5.12	42.5	/	/	10 legs, in‐plane	40	127.9	4.9 × 10^−4^	/	/	Flexible	[34]
Pb_x_(Bi_0.5_Sb_1.5_)_1−x_Te_3_ thread	p	300	240	6 ± 0.8	34.56	0.25	0.04	5 p‐n pairs, in‐plane	10	8.5	2.6 × 10^−5^	/	/	Flexible	[84]
		360	260	9.6	65	0.24	0.08								
Bi_2_Te_2.73_Se_0.3_ thread	n	300	120	1.7 ± 0.6	2.45	0.25	0.003								
		360	140	1.8	3.5	0.21	0.005								
Carbon black /Bi_0.4_Sb_1.6_Te_3_/PLA film	p	300	119.9	13.3	19.2	0.25	0.023	/	/	/	/	/	/	Flexible	[79]
Bi_0.4_Sb_1.6_Te_3_/PLA‐Ag/PLA double‐layer film	p	300	80	1170	748.8	/	/	/	/	/	/	/	/	Flexible	[80]
MoS_2_/PEDOT:PSS film	p	300	12.7	30.15	0.49	/	/	/	/	/	/	/	/	Flexible	[32]
Black phosphorus/PEDOT:PSS film	p	300	12	833.33	12	/	/	/	/	/	/	/	/	Flexible	[78]
		340	13.2	763.31	13.3	/	/								
3D TPU/carbon nanofibers sponge	p	298	30.8	/	/	/	/	/	/	/	/	/	/	Flexible	[43]
SWCNT/PAA film	p	/	48	559.9	129	/	/	60 p‐n pairs, out‐of‐plane	30	130	1.95× 10^−3^	/	/	Flexible	[35]
SWCNT/PEI film	n	/	33	1239.7	135	/	/								
SWCNT/PAA film	p	300	63.4	810	325.58	/	/	140 p‐n pairs, out‐of‐plane	20	188.08	1.68 × 10^−3^	/	/	Flexible	[33]
SWCNT/PEI film	n	300	35	2260	276.85	/	/								
SWCNT/PAA film	p	300	59.19	797.04	279.24	/	/	/	/	/	/	/	/	Flexible	[41]
SWCNT/PEI film	n	300	27.59	1794.81	136.62	/	/								
PEDOT:PSS/MWCNT film	p	298	22.8	185.19	7.37	/	/	5 legs, in‐plane	50	6.4	40.48 × 10^−6^	/	/	Flexible	[95]
PEDOT:PSS‐SWCNTs/epoxy resin block	p	303	28.5	520	42.23	0.175	0.07	2 p−n pairs, out‐of‐plane	100	13.6	4.1 × 10^−3^	Cuboidal leg of 2 mm × 2 mm	256	Flexible	[36]
		453	41	620	102	0.09	0.52								
PEI‐SWCNTs/epoxy resin block	n	303	27.5	370	28	0.135	0.06								
		453	40	471	75	0.07	0.47								
Bi_0.4_Sb_1.6_Te_3_ half ring	p	303	170	520	1502.8	0.6	0.75	3 p−n pairs	39.2	27.0	1.62	Half‐rings with d = 8 mm, D = 15 mm, T = ≈1.53 mm	1.42 × 10^4^	Conformal	[73]
		398	197	326	1265.17	0.55	0.9								
Bi_2_Sb_2.7_Se_0.3_ half ring	n	303	120	500	720	0.55	0.39								
		443	145	372	782.13	0.5	0.6								
Pb_0.98_Na_0.02_Te tube	p	300	50	2000	500	3	0.05	5 p–n pairs	300	83.2	216.3		1.537 × 10^6^	Conformal	[39]
		700	250	316	1975	0.99	1.4								
Pb_0.98_Sb_0.02_Te tube	n	300	60	1400	504	1.85	0.08								
		700	177	350	1096.5	0.63	1.2								
Bi_0.5_Sb_1.5_Te_3_/PVP annular ring	p	298	149	80	177.68	0.543	0.104	5 p–n pairs	54.6	60.8	0.68	/	/	Conformal	[81]
		573	120	48	69	0.525	0.07								
Bi_2_Te_3_/PVP annular ring	n	298	135	103.3	188.1	0.579	0.11								
		573	115	55	73	0.545	0.08								
PAA‐PEI‐Bi_0.5_Sb_1.5_Te_3.0_ half annular ring	p	300	144.1	770	1600	0.97	0.49	3 p–n pairs	55.6	40.61	0.38	/	/	Conformal	[37]
		475	≈163	≈560	≈1488	≈1.0	0.71								
PAA‐PEI‐Bi_2.0_Te_2.7_Se_0.3_ half annular ring	n	300	148.1	515	1130	0.81	0.42								
		450	≈165	≈390	≈1062	≈0.8	0.59								
PAA‐PEI‐Bi_0.5_Sb_1.5_Te_3.0_/MC half ring	p	300	≈160	≈574	≈1469	≈0.83	0.53	3 p–n pairs	55.1	42.25	0.45	/	/	Conformal	[87]
		450	169	398	1136.73	0.79	0.65								
PAA‐PEI‐Bi_2.0_Te_2.7_Se_0.3_/MC half ring	n	300	≈160	≈407	≈1042	≈0.77	0.41								
		425	172	305	902.31	0.72	0.53								
PEDOT:PSS thread	p	300	16–17	5.85 ± 0.1	0.11–0.16	/	/	One p–n pair, in‐plane	120	2.1	5.17 × 10^−6^	/	/	None	[64]
Cu_2_Se honeycomb‐based TE leg	p	1000	186	174.9	605	0.5	1.21	One single leg, out‐of‐plane	509.7	52	121	Cross‐sectional area of 105.21 mm^2^	6.21 × 10^6^	None	[31]
Vertical Bi_0.55_Sb_1.45_Te_3_ filament	p	300	191.2	650	2376.2	0.85	0.84	3 p–n pairs, out‐of‐plane	82.9	42.4	2.8 × 10^−3^	Filaments of d = 0.35 mm and h = 1.4 mm	4.79 × 10^3^	None	[75]
Vertical Bi_2_Te_2.7_Se_0.3_ filament	n	300	111.7	792	988.2	0.8	0.37								
Bi_0.45_Sb_1.55_Te_3_ cube	p	298	166.9	980	2730	1.0	0.81	One segmented leg, out‐of‐plane	236.1	56.7	260.4	10 mm × 10 mm (L × W)	2.6 × 10^6^	None	[76]
3D Bi_2_Te_3_ nanowire structures	n	303	112.8	711	904.3	1.37	0.2	/	/	/	/	/	/	None	[94]
3D Bi_0.5_Sb_1.5_Te_3_/Pluronic F127 structure	p	433	203	9.05	37.3	0.09	0.187	/	/	/	/	/	/	None	[82]
Bi_0.4_Sb_1.6_Te_3_+7%Te strip	p	309	213	279	1266	/	/	/	/	/	/	/	/	None	[83]
Bi_2_Te_2.6_Se_0.4_+10%Se strip	n	313	159.3	316	802	/	/	/	/	/	/	/	/	None	[83]
3D TiNiSn structure	n	325	80	/	/	/	/	/	/	/	/	/	/	None	[86]
		698	183	/	/	/	/								
Cu_2_Se hourglass‐shaped TE leg	p	298	≈86	≈580	≈428	≈0.37	≈0.35	One single leg, out‐of‐plane	500	≈72	≈26	/	/	None	[96]
(Bi, Sb)_2_Te_3_ rectangular‐shaped TE leg	p	303	258	148	985	0.21	1.42	/	/	/	/	/	/	None	[42]
Ag_2_Se rectangular‐shaped TE leg	n	303	175	586	1794.6	2.4	1.3								

Noting that *T*, *S*, *σ*, *PF*, *κ*, and *zT* stand for the measuring temperature (K), Seebeck coefficient (µV K^−1^), electrical conductivity (S cm^−1^), power factor (µW m^−1^ K^−2^), thermal conductivity (W m^−1^ K^−1^), and figure‐of‐merit for TE materials, respectively. Furthermore, Δ*T* stands for temperature differences (K) between the hot and cold sides of TEGs, *V*_oc_, *V*_output_, *P*_max_, and *Pd*_max_ stand for open‐circuit voltage (mV), output voltage (mV), maximum output power (mW), and maximum output power density (mW m^−2^) of TEGs, respectively. The units of various parameters in the literature have been normalized. ‘/’ represents the absence of certain substance or preparation conditions that are not explicitly stated in the literature. *T* was unified with the conversion formula *T* (K) = *x* (°C) + 273, and the room temperature mentioned in the literature was set at 25°C.

Nevertheless, the conversion efficiency of TEDs fabricated via solution 3D printing is generally lower than that of devices produced using traditional methods such as zone melting or spark plasma sintering. The main reasons are as follows: 1) Inherently low thermoelectric performance of organic and carbon‐based materials: TEDs printed with such inks typically exhibit low conversion efficiency. 2) Interference from organic additives in all‐inorganic or hybrid inks: To ensure ink stability and printability, polymer binders (e.g., PVP) or dispersants (e.g., glycerol) are often required. After sintering, residual organics or carbonaceous by‐products remain at grain boundaries, forming potential barriers that severely impede charge transport. 3) Non‐dense microstructures: Printed lines frequently contain pores and cracks, which reduce the effective conductive cross‐sectional area and increase electrical resistance. 4) Small grain size: Powders used in inks and grains formed during post‐printing heat treatment are typically fine, leading to a high density of grain boundaries that scatter charge carriers and lower mobility. Collectively, points (1)–(4) contribute to reduced electrical conductivity and a compromised Seebeck coefficient. 5) Interface challenges: At the device level, poor connections between thermoelectric legs and electrodes or substrates can result in high contact resistance and weak adhesion, causing excessive Joule heat losses and diminished output power.

To address the above challenges, several targeted strategies are being actively explored: 1) Solvent post‐treatment: This approach is widely applied to remove insulating residues from printed thermoelectric materials, thereby improving electrical transport and overall performance.^[^ ^30^, ^65^ ^]^ 2) Hybrid inks: Incorporating multiple thermoelectric active components generates heterogeneous grain boundaries and interfaces, which can significantly influence charge carrier transport.^[^ ^79^ ^]^ 3) Nanostructure engineering: For inorganic thermoelectric systems, precise control of annealing or sintering conditions (e.g., temperature, time, atmosphere) can regulate grain growth while preserving nanoscale porosity and introducing multi‐scale carrier scattering centers within the printed structure.^[^ ^31^, ^42^ ^]^ 4) Integrated printing: Directly printing thermoelectric legs together with inter‐electrodes minimizes contact resistance, which typically increases during stepwise assembly, thereby improving device output power.^[^ ^33^, ^36^, ^40^ ^]^ Conformal architectures: Printing thermoelectric legs conformally onto circular or curved surfaces (e.g., pipes, engine casings) enables the construction of conformal TEDs that maximize the heat source surface area, increase the number of thermoelectric legs, and consequently enhance total output power.

## 5 Application of Solution 3D Printed TEs

### 5.1 TE Power Generation

Solution 3D‐printed TEGs fabricated on flexible substrates can conform to curved surfaces and accommodate dynamic human movements, enabling efficient thermal energy harvesting from temperature gradients between the skin and the ambient environment. Their high adaptability and intimate contact with the heat source minimize thermal losses, thereby enhancing thermoelectric conversion efficiency.

**Figure 17:**
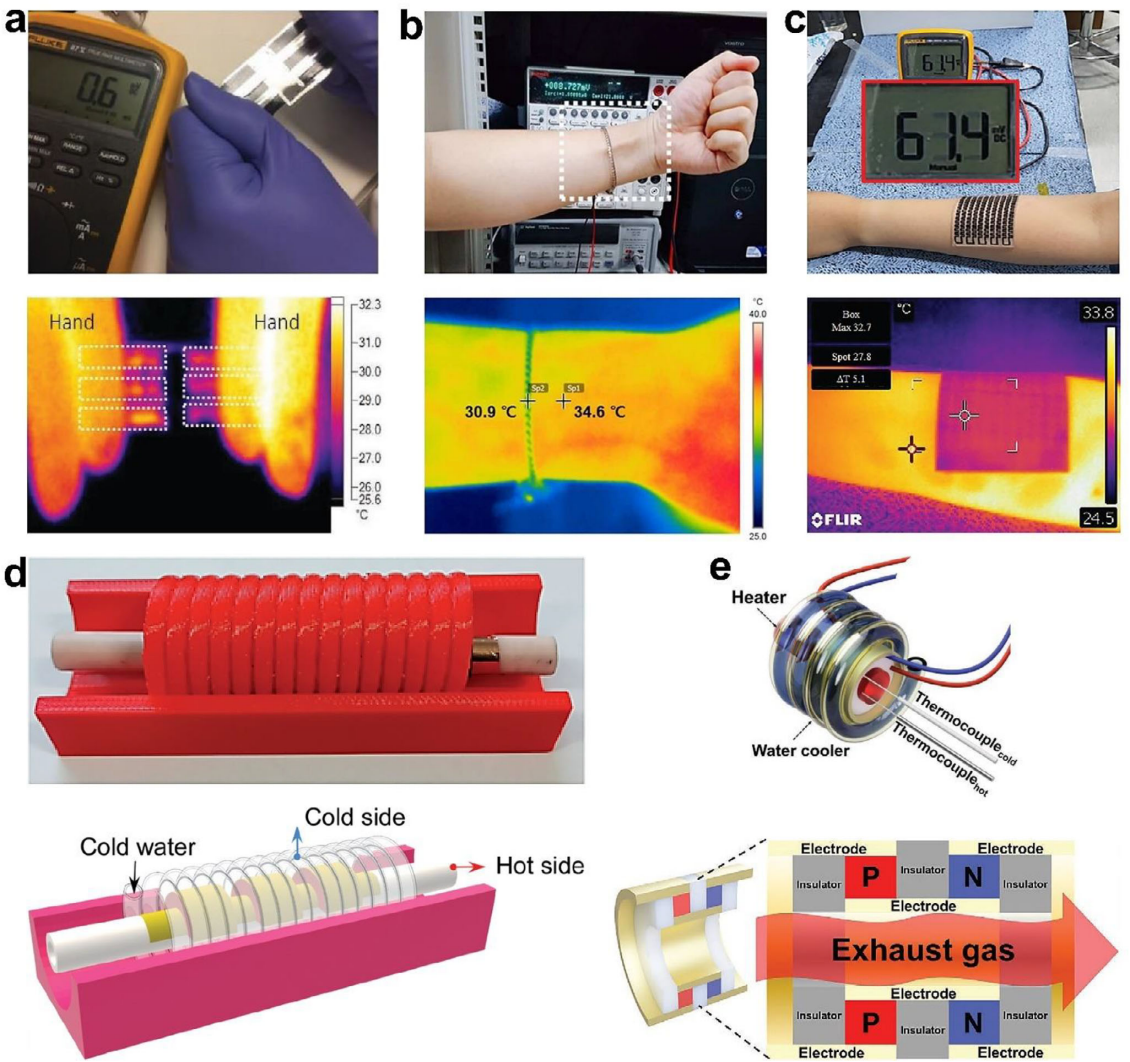
Application examples of thermoelectric power generation for solution 3D printed TEDs. a–c) Demonstrations of human body temperature‐based thermoelectric power generating. Reproduced with permission.^[^ ^38^ ^]^ Copyright 2019, Wiley‐VCH. Reproduced with permission.^[^ ^35^ ^]^ Copyright 2018, Royal Society of Chemistry. Reproduced with permission.^[^ ^33^ ^]^ Copyright 2023, Wiley‐VCH. d,e) Photograph and scheme for potential application of conformal TEGs attached onto heating pipes. Reproduced with permission.^[^ ^37^ ^]^ Copyright 2022, Royal Society of Chemistry. Reproduced with permission.^[^ ^39^ ^]^ Copyright 2021, Wiley‐VCH.

As shown in **Figure** **17**a, a 6‐leg PEDOT:PSS/Triton X‐100 film‐based TEG generated a stable *V*_output_ of 0.6 mV under a Δ*T* of ≈7 K induced by human finger contact at ambient temperature, closely matching the theoretical value.^[^ ^38^ ^]^ Figure 17b displays a bracelet‐type TEG with CNT‐based legs (30 p–n pairs), which produced a *V*_output_ of 9 mV at a Δ*T* of 3.7 K.^[^ ^35^ ^]^ Based on the *S* of its *p*‐type (48 µV K^−1^) and n‐type (33 µV K^−1^) thermoelectric legs, the theoretical voltage was calculated to be 8.99 mV, confirming efficient thermal energy utilization and strong potential for wearable energy harvesting. Similarly, the 3D‐compliant TEG (Figure 17c), consisting of 140 CNT‐based p–n pairs printed on a flexible PDMS substrate, was designed for direct application on the human body. When conformally worn on the forearm, it generated a *V*_output_ of 61.4 mV at a Δ*T* of 5.1 K, achieving ≈95% of the theoretical value.^[^ ^33^ ^]^

Unlike flexible printed TEGs designed to accommodate arbitrary shapes, conformal TEGs are typically tailored for specific surfaces, with inorganic‐based thermoelectric couples directly printed for targeted applications, e.g., industrial heating pipelines.^[^ ^37^, ^39^, ^73^, ^81^, ^87^ ^]^ Figure 17d shows a prototype photograph and power generation scheme of the conformal TEG composed of Bi_2_Te_3_‐based half‐annular‐rings.^[^ ^37^ ^]^ The internal and external ends of the half‐annular‐rings were respectively adhered to Cu inter‐electrodes using Ag epoxy paste. As the inner side of the conformal TEG tightly stuck on the alumina pipe, the temperature differences established between the hot alumina pipe and cooling pipeline could be efficiently utilized, thus efficient generation of electrical power was achieved. Likewise, for conformal TEGs assembled from circular or tubular modules, they could generate electrical power by clamping onto pipelines transporting waste heat as indicated in Figure 17e.^[^ ^39^ ^]^ These designs highlight their potential for efficient waste heat recovery in industrial environments.

### 5.2 Sensing

When subjected to a temperature gradient, the *V*_oc_ of a single printed thermoelectric leg or an entire printed device can serve as a sensing signal, enabling the use of these thermoelectrics as thermoelectric‐powered temperature sensors. Moreover, certain flexible printed thermoelectrics with excellent structural recoverability can support dual‐mode sensing capabilities. These multifunctional sensors hold significant promise for applications in high‐temperature/fire warning systems, healthcare monitoring, prosthetics, and human‐machine interfaces.

**Figure 18:**
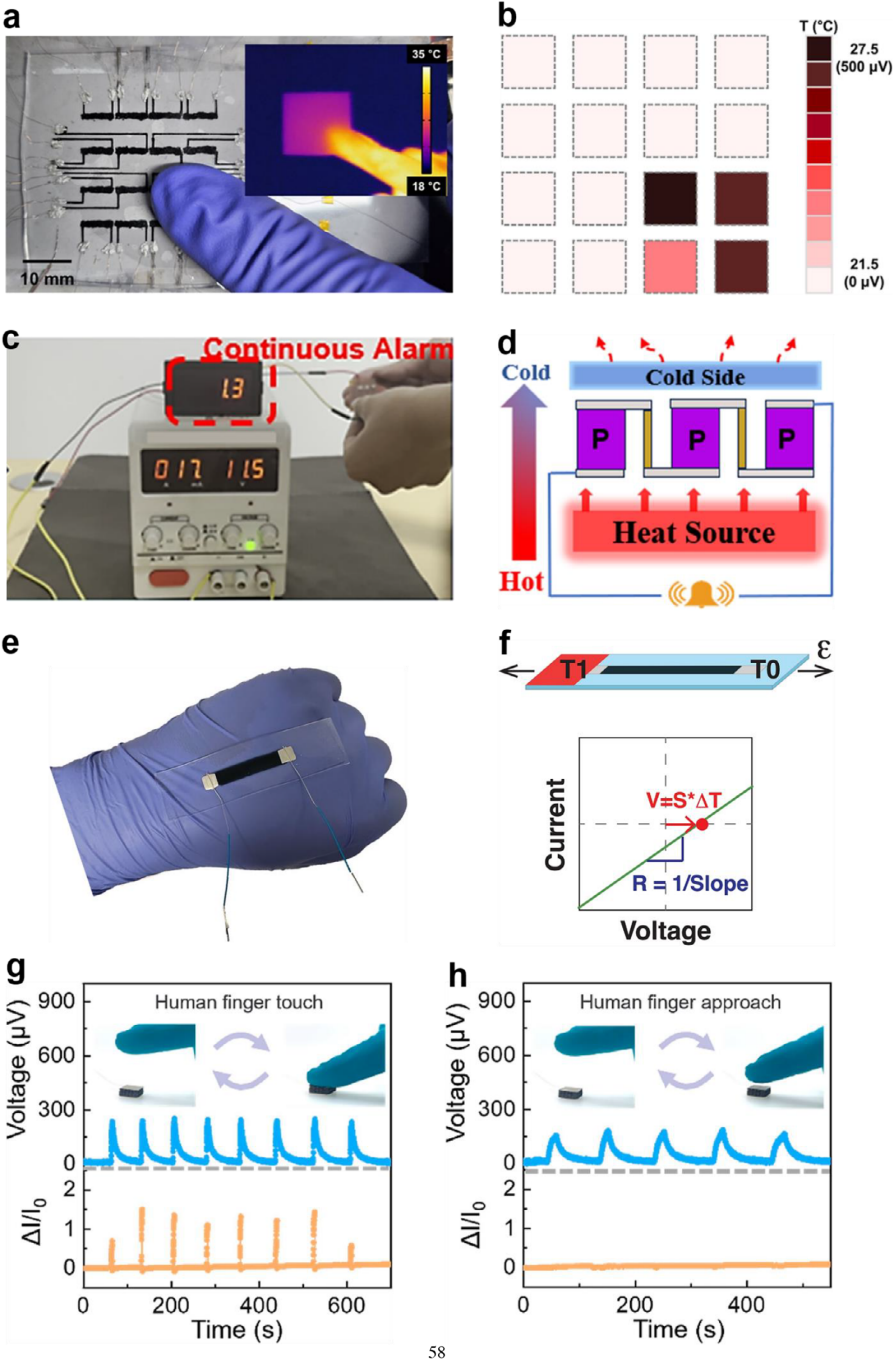
Application examples of sensing for solution 3D printed TEDs. a,b) Localized temperature detection by the 4 × 4 array temperature sensor based on CNTs. Reproduced with permission.^[^ ^41^ ^]^ Copyright 2024, American Chemical Society. c,d) Touch testing of a TEG‐based temperature alarm response device based on Bi_2_Te_3_. Reproduced with permission.^[^ ^34^ ^]^ Copyright 2024, American Chemical Society. e,f) Simultaneous strain and temperature sensing of PEDOT:PSS‐based sensors. Reproduced with permission.^[^ ^40^ ^]^ Copyright 2024, IEEE. g,h) Current and voltage responses to the “human finger touch” and “human finger approach” of a flexible pressure‐temperature sensor based on TPU/CNFs sponge. Reproduced with permission.^[^ ^43^ ^]^ Copyright 2021, Elsevier. Copyright 2024, IEEE.

For single‐mode sensing applications, the all‐in‐one temperature sensor array printed from CNT‐based solutions (**Figure** **18**a) was capable of accurately detecting the temperatures of four individual pixels covered by human fingers.^[^ ^41^ ^]^ Additionally, when connected in series with an alarm system, the temperature‐responsive device (Figure 18b) enabled wide‐range thermal sensing by adjusting the voltage threshold required to trigger the alarm sound.^[^ ^34^ ^]^ Both of these thermoelectric‐powered temperature sensors demonstrate strong potential for hot‐spot detection and localized temperature monitoring.

For dual‐mode sensing applications, a single sensor based on a printed PEDOT:PSS–PEO composite film (Figure 18c) can simultaneously detect both strain and temperature stimuli. While the *S* of the thermoelectric film remains stable, resistance changes under mechanical deformation are reflected by variations in the slope of the output signal at a given Δ*T*.^[^ ^40^ ^]^ Similarly, the flexible pressure–temperature sensor based on a TPU/CNF sponge (Figure 18d) enables complete decoupling of pressure and temperature sensing. It maintains a stable thermoelectric response under compressive strain and exhibits negligible resistance variation due to temperature, ensuring accurate and independent dual‐mode sensing.^[^ ^43^ ^]^

### 5.3 Active Coolin

Despite recent advances in solution 3D printing of thermoelectric materials, their application in active cooling has remained challenging due to limited efficiency, often attributed to the presence of organic components or non‐densified alloy structures. Recently, Xu et al.^[^ ^42^ ^]^ addressed these limitations by optimizing ink formulations and assembling a TEC using directly printed p‐type and n‐type pillars, in which interfacially bonded networks were formed. For the binder‐free *n*‐type Ag_2_Se ink, grain bonding was achieved through a phase transition at ≈133 °C during sintering. For the *p*‐type (Bi, Sb)_2_Te_3_ ink, formulated with Bi nanoparticles and Sb_2_Te_4_ ChaM binders, a new (Bi, Sb)_2_Te_3_ phase formed during sintering, bonding the original particles. This study represents the first detailed investigation of the cooling performance and stability of a solution 3D‐printed TED, marking a significant step toward practical cooling applications.

**Figure 19:**
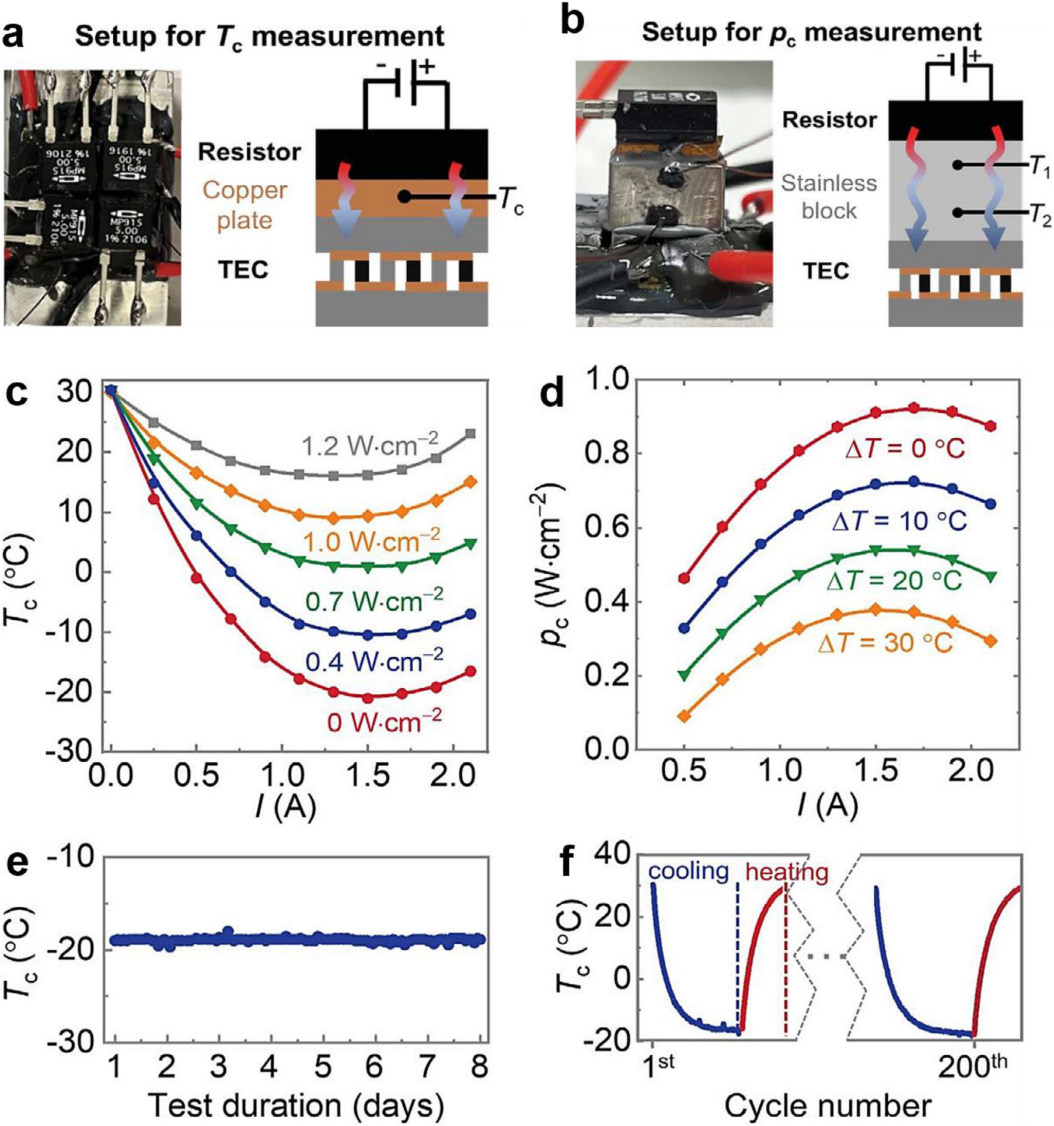
Application examples of active cooling for solution 3D printed TEDs. Cooling performance and stability of the 3D‐printed TEC based on p‐type (Bi, Sb)_2_Te_3_ pillars and n‐type Ag_2_Se pillars, including a) home‐made setups for cooling temperature *T*_c_ and b) cooling flux density *p*_c_ measurements, c) *T*_c_ as a function of heating loads on the cold surface of the TEC, d) *p*_c_ as a function of different Δ*T* across the TEC, e) *T*_c_ of the TEC during continuous cooling test for 7 days, and f) *T*_c_ of the TEC during cyclic cooling tests. Reproduced with permission.^[^ ^42^ ^]^ Copyright 2025, American Association for the Advancement of Science.

As shown in **Figure** **19**a,b, the 32‐pair TEC was evaluated using custom‐built setups to measure the *T*_c_ and cooling flux density (*p*_c_). Without an applied heating load, the device achieved a maximum cooling temperature difference (Δ*T*_max_) of up to 50 °C in air, with the hot side maintained at 30 °C (Figure 19c). It also demonstrated a peak cooling flux density (*p*_c,max_) of 0.87 W cm^−2^ under zero Δ*T* conditions (Figure 19d). The directly printed TEC exhibited excellent reliability and operational stability, with *T*_c_ remaining constant after a continuous 7‐day cooling test (Figure 19e) and 200 thermal cycling tests (Figure 19f). These results underscore the potential of solution 3D printing as a sustainable and efficient manufacturing platform for thermoelectric devices.

## 6 Conclusion and Outlook

This study presents a comprehensive overview of recent advancements in thermoelectric materials and devices fabricated via solution 3D printing. It covers the development of various thermoelectric ink compositions, material and device configurations, reported performance metrics, and practical application potentials. This review aims to provide researchers with an in‐depth understanding of current progress in the field and to inspire future innovations in the design and fabrication of high‐performance, flexible, and adaptable TEDs.

Solution 3D printing offers notable advantages for thermoelectric device fabrication, including pattern customizability, integrated shaping, and scalable manufacturing. However, several challenges remain to be addressed to fully realize its potential. All‐organic thermoelectric inks are low‐cost, non‐toxic, and environmentally friendly, and offer excellent printability. The resulting thermoelectric materials and devices exhibit good flexibility and wearability but are limited by relatively low thermoelectric performance. In contrast, all‐inorganic thermoelectric inks often deliver superior thermoelectric properties, broader temperature applicability, and structural stability, but involve toxic elements (e.g., Te, Pb, Cd), high material consumption, complex preparation procedures, fine‐vacuum environments, and high‐temperature post‐treatments such as annealing. These factors result in stiff and heavy devices with limited flexibility and wearability. Organic–inorganic hybrid inks, which combine the flexibility of polymers with the high thermoelectric performance of inorganic fillers, represent the most widely adopted approach for producing high‐performance flexible thermoelectric materials. These hybrid systems achieve a balance between thermoelectric performance and mechanical compliance. Nevertheless, challenges remain, including poor control over the organic–inorganic interface and the difficulty in maintaining slurry dispersibility and long‐term stability. CNT‐based inks, owing to their 1D morphology and high aspect ratio, offer excellent printability and tuneable thermoelectric performance via doping. They enable fully printed thermoelectric legs and interconnects, eliminating the need for metal leads and conductive pastes, thereby improving interface compatibility and mechanical flexibility. However, their application is hindered by poor dispersion stability. Future research should aim to overcome these limitations, particularly in material formulation, interface engineering, and processing techniques, to unlock the full potential of solution 3D printing for next‐generation thermoelectric materials and devices.

Printed thermoelectric materials and devices have been successfully applied in TEGs, temperature sensors, stress–strain sensors, and dual‐mode pressure–temperature sensors. However, challenges remain for TEDs fabricated via solution 3D printing. In flexible TEDs, the use of insulating film substrates hinders heat conduction, as the heat flow is directed through the out‐of‐plane axis of 2D thermoelectric modules, thereby reducing thermal energy utilization efficiency. For conformal TEDs, the post‐printing assembly process is often complex and labor‐intensive. Moreover, these devices typically exhibit limited cooling capacity due to the presence of residual organic components and the insufficient densification of printed thermoelectric structures. In addition, printed thermoelectric pillars tend to exhibit high internal electrical resistance, leading to significant Joule heating that can surpass the Peltier cooling effect, further limiting active cooling performance.

To overcome these challenges, increased attention and dedicated efforts are needed to address the remaining technical limitations and advance key research areas. Achieving meaningful progress will require sustained, interdisciplinary collaboration among experts in chemistry, physics, materials science, and engineering. The outlooks are enumerated as follows:

1. One‐step synthesis of thermoelectric slurries. Most existing thermoelectric slurries are prepared via stepwise mixing, which often leads to uneven dispersion of active thermoelectric components within the adhesive matrix, ultimately compromising the performance of the printed materials and devices. A one‐step synthesis approach could address these issues by enabling complete dissolution of thermoelectric precursors within the adhesive medium. By introducing appropriate catalysts or reducing agents, this method can produce homogeneous slurries with improved compositional uniformity and enhanced functional performance.
2. Material‐centered performance optimization. On one hand, the intrinsic thermoelectric properties and dispersibility of active thermoelectric components should be further optimized to enable the fabrication of printed products with enhanced performance, even when using insulating binders or solvents. On the other hand, post‐processing techniques, such as solution impregnation and thermal annealing, should be further developed or integrated to mitigate the negative effects of adhesive residues. These treatments can enhance thermoelectric performance while preserving the structural integrity and geometric precision enabled by 3D printing.
3. Flexible out‐of‐plane structure design. Currently, most in‐plane flexible TEDs with favorable mechanical and thermoelectric performance benefit from the use of inorganic thermoelectric components in the printing slurries and the flexibility of the supporting substrates. However, to overcome limitations in performance testing and practical deployment, flexible out‐of‐plane device architectures should be developed. These structures would allow for direct Δ*T* between the heat source and the environment, simplifying integration and enhancing application potential.
4. Exploration of new material systems and application possibilities. Priority should be given to the in‐depth exploration of a broader range of printable materials with specialized properties, such as high mechanical strength, electrical transport properties, and biocompatibility. Simultaneously, advancements in solution 3D printing technology are essential to better accommodate the diverse physical and chemical characteristics of these materials. Expanding the range of printable materials, alongside the development of a more adaptable and robust printing process, will open new possibilities for applications across a wider array of fields (Supporting Information).

## Conflict of Interest

The authors declare no conflict of interest.

## Author Contributions

M.D. performed investigation, wrote the original draft, and reviewed and edited the final manuscript. X.L.S. wrote, review and edited the final manuscript. Y.D., T.Z. and Z.‐G.C. contributed to the conceptualization, supervision, and wrote, reviewed and edited the final manuscript.

## Supporting information



Supporting Information

## Data Availability

Data will be made available on request.
