# Supplementary material for: Solution‐Based 3D Printing of Thermoelectrics: Advances and Opportunities
Source: Adv Sci (Weinh). 2025 Nov 3;12(46):e14259. doi: 10.1002/advs.202514259 (PMC12697775; doi:10.1002/advs.202514259)
Supplement: Supplementary file 1 — Supporting Information [file ADVS-12-e14259-s001.docx]

# Appendix 1. Abbreviation summery

Abbreviations of elemental materials, solvents, polymers, *etc.* that appear in the main text were summarized as follows.

**Table S1.** Abbreviation of elemental materials

| **Abbr.** | **Element** |  | **Abbr.** | **Element** |  | **Abbr.** | **Element** |  | **Abbr.** | **Element** |
| --- | --- | --- | --- | --- | --- | --- | --- | --- | --- | --- |
| Ag | Silver |  | Bi | Bismuth |  | Sb | Antimony |  | Co | Cobalt |
| Au | Gold |  | Te | Tellurium |  | Nb | Niobium |  | Fe | Ferrum |
| Pt | Platinum |  | Se | Selenium |  | Pb | Lead |  | Na | Sodium |
| Ar | Argon |  | N | Nitrogen |  | Cd | Cadmium |  | Ti | Titanium |
| Ni | Nickel |  | Sn | Tin |  |  |  |  |  |  |

**Table S2.** Abbreviation of solvents

| **Abbr.** | **Solvent** |  | **Abbr.** | **Solvent** |
| --- | --- | --- | --- | --- |
| EG | Ethylene glycol |  | DMSO | Dimethyl sulfoxide |
| CLM | Chloroform |  | DCM | Dichloromethane |
| DEG | Diethylene glycol |  | NMF | N-methylformamide |
| BCS | 2-butoxy ethanol |  | DMF | Dimethyl formamide |
| EGBE | Ethylene glycol butyl ether |  | DBP | Dibutyl phthalate |

**Table S3.** Abbreviation of polymers

| **Abbr.** | **Polymer** |  | **Abbr.** | **Polymer** |
| --- | --- | --- | --- | --- |
| PLA | Polylactic acid |  | PLG | Polylactide-co-glycolide |
| TPU | Thermoplastic polyurethane |  | PVP | Polyvinyl pyrrolidone |
| PU | Polyurethane |  | PDMS | Polydimethylsiloxane |
| PAA | Poly(acrylic acid) |  | PEI | Poly(ethylenimine) |
| SAP | Superabsorbent-polymer |  | PS | Polystyrene |
| MC | Methylcellulose |  | PEO | Poly(ethylene oxide) |
| PPO | Poly(propylene oxide) |  | GOPS | (3-glycidyloxypropyl)trimethoxysilane |
| Pluronic F127 | PEO-PPO-PEO copolymer |  | ABS | Acrylonitrile butadiene styrene |
| PEN | Polyethylene naphthalateare |  | PET | Polyethylene terephthalate |
| PI | Polyimide |  |  |  |
